# RNA Sequence Analyses throughout the Course of Mouse Cardiac Laminopathy Identify Differentially Expressed Genes for Cell Cycle Control and Mitochondrial Function

**DOI:** 10.1038/s41598-020-63563-x

**Published:** 2020-04-20

**Authors:** Zhili Shao, Wonshill Koh, Ying Ni, Wei Li, Brendan Agatisa-Boyle, Daria Merkurjev, Wai Hong Wilson Tang

**Affiliations:** 1 Department of Cardiovascular and Metabolic Sciences, Lerner Research Institute, Cleveland Clinic, Cleveland, OH USA; 2 Department of Cardiology, Cincinnati Children’s Hospital Medical Center, Cincinnati, OH USA; 3 Department of Quantitative Health Sciences, Lerner Research Institute, Cleveland Clinic, Cleveland, OH USA; 4 Department of Biomedical Sciences, Joan C. Edwards School of Medicine, Marshall University, Huntington, WV USA; 5 Division of Cardiology, Department of Medicine, University of California at Los Angeles, Los Angeles, CA USA; 6 Kaufman Center for Heart Failure Treatment and Recovery, Department of Cardiovascular Medicine, Heart, Vascular & Thoracic Institute, Cleveland Clinic, Cleveland, OH USA

**Keywords:** Cardiology, Cardiovascular diseases, Gene expression

## Abstract

Lamin A/C (*LMNA*) gene mutations are a known cause of familial dilated cardiomyopathy, but the precise mechanisms triggering disease progression remain unknown. We hypothesize that analysis of differentially expressed genes (DEGs) throughout the course of *Lmna* knockout (*Lmna*^−/−^)-induced cardiomyopathy may reveal novel *Lmna*-mediated alterations of signaling pathways leading to dilated cardiomyopathy. Although *Lmna* was the only DEG down-regulated at 1 week of age, we identified 730 and 1004 DEGs in *Lmna*^−/−^ mice at 2 weeks and 1 month of age, respectively. At 2 weeks, *Lmna*^−/−^ mice demonstrated both down- and up-regulation of the key genes involving cell cycle control, mitochondrial dysfunction, and oxidative phosphorylation, as well as down-regulated genes governing DNA damage repair and up-regulated genes involved in oxidative stress response, cell survival, and cardiac hypertrophy. At 1 month, the down-regulated genes included those involved in oxidative phosphorylation, mitochondrial dysfunction, nutrient metabolism, cardiac β-adrenergic signaling, action potential generation, and cell survival. We also found 96 overlapping DEGs at both ages involved in oxidative phosphorylation, mitochondrial function, and calcium signaling. Impaired oxidative phosphorylation was observed at early disease stage, even before the appearance of disease phenotypes, and worsened with disease progression, suggesting its importance in the pathogenesis and progression of *LMNA* cardiomyopathy. Reduction of oxidative stress might therefore prevent or delay the development from *Lmna* mutation to *LMNA* cardiomyopathy.

## Introduction

Lamin A/C (*LMNA*) gene mutations account for approximately 6–8% of known genetic dilated cardiomyopathies, and occur with frequent cardiac conduction system disease^1^. Lamins are type V intermediate filament proteins and major components of the nuclear lamina. They line the inner surface of the inner nuclear membrane, where they interact with chromatin associated proteins, nuclear envelope proteins (including nuclear pore complexes), and transcription factors, thereby regulating important cellular events such as chromatin organization, DNA repair/replication, transcription and cell division^2–5^. There are two types of lamins. Lamins A and C are A-type lamins coded by the *Lmna* gene, while B-type lamins (B1 and B2) are coded by two different genes (*Lmnb1* and *Lmnb2*, respectively). Unlike B-type lamins, lamins A and C are also found in nucleoplasm and may play a role in regulating the expression of certain genes^5,6^. Much effort has been undertaken to identify signaling molecules implicated in *Lmna* mutations, and especially in *LMNA* cardiomyopathy, in order to gain insights on how lamins regulate a wide spectrum of cellular processing leading to cardiomyopathy and heart failure^7–16^. Previously, microarray analysis in a mouse model harboring the *Lmna* mutation (*Lmna*^*H222P/H222P*^) for Emery-Dreifuss muscular dystrophy and cardiomyopathy showed that there was over-activation of mitogen-activated protein kinase 3/1 (ERK1/2) and mechanistic targeting of rapamycin kinase (mTOR) pathways^7–9,17,18^. Therapeutic inhibition of these pathways has shown delayed left ventricular dilation and improved cardiac function in the animal model of *LMNA* cardiomyopathy^8,10–14,18^. Recent RNA sequencing studies found the activation of FOXO transcription factors (in 2-week-old *Lmna*^−/−^ mice) and E2F/DNA damage response/TP53 pathway (in 2-week *Lmna*^*D300N*^ mice) contribute to the pathogenesis of *LMNA* cardiomyopathy^15,16^.

Compared to microarrays, RNA-Sequencing technology produces discrete, digital sequencing read counts and can quantify expression across a larger dynamic range (>10^5^ versus 10^3^ for arrays) and detect a higher percentage of differentially expressed genes. This is especially true for genes with low expression^19–21^. In our present study, we utilized RNA sequence analysis to investigate gene expression profiling throughout the lifespan of *Lmna*^−/−^ mice with cardiomyopathy to investigate novel *Lmna*-mediated alterations of signaling pathways leading to dilated cardiomyopathy. While previous studies focused on specific pathways^8,10–16,18^ in the pathogenesis of *LMNA* cardiomyopathy (caused by specific *Lmna* mutations or knockout), our study aimed to provide a systematic overview of the signaling pathways and pathophysiological changes that might synergistically contribute to the development of *Lmna*^*−/−*^ induced *LMNA* cardiomyopathy. To the best of our knowledge, this is the first systematic mechanism study covering the entire disease process of *LMNA* cardiomyopathy in the *Lmna*^−/−^ mouse model. Rather than clarifying a specific pathogenic signaling pathway, this study reveals key signaling pathways involved in the pathogenesis of *LMNA* cardiomyopathy and provides guidance for further mechanism studies. In particular, our study is the first to identify the importance of impaired oxidative phosphorylation in the pathogenesis and progression of *LMNA* cardiomyopathy, suggesting the possibility that a reduction in oxidative stress might prevent or delay the development of LMNA cardiomyopathy in the presence of a *Lmna* gene mutation.

## Results

### Homozygous *Lmna*^−/−^ mice exhibit severe growth retardation and cardiac dysfunction at 1 month of age

**Figure 1:**
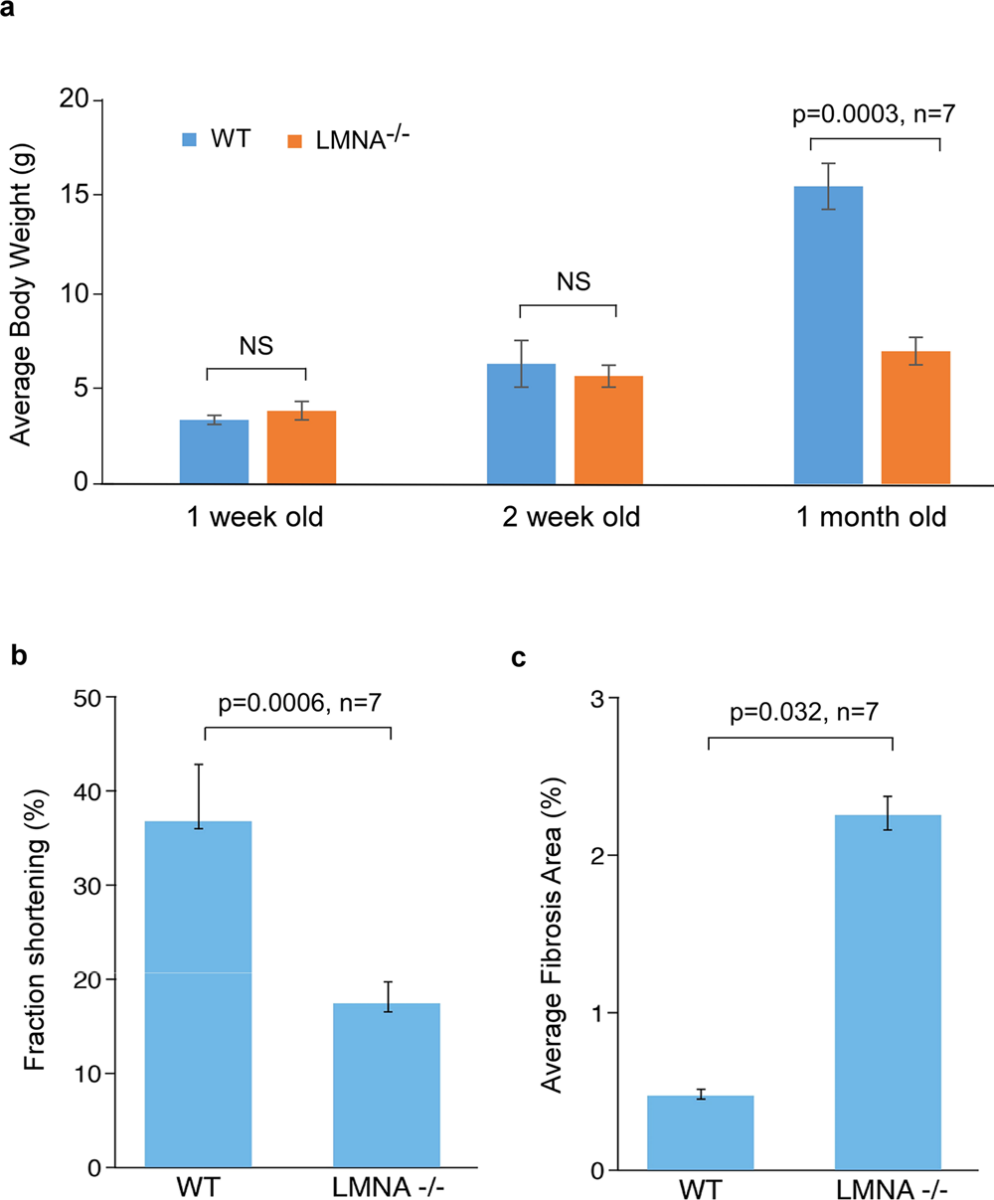
*Lmna*^−/−^ Mice Exhibited Severe Growth Retardation (A), Cardiac Dysfunction (B) and Increased Myocardial Fibrosis (C) at One Month of Age. **(a)** Significantly lower body weight was found in 1-month-old *Lmna*^−/−^ mice compared with 1-month-old WT mice (p = 0.0003, n = 7 for each group). There was no statistical difference in body weight between *Lmna*^−/−^ and WT mice at 1 week or 2 weeks old. **(b)** 1-month-old *Lmna*^−/−^ mice demonstrated a significantly less fractional shortening percentage than 1-month-old WT mice (p = 0.0006, n = 7 for each group). **(c)** Picrosirus Red staining of left ventricular myocardium showed an increased ratio of fibrotic areas to the whole myocardium in 1-month-old *Lmna*^−/−^ mice compared to 1-month old WT mice (p = 0.032, n = 7).

Homozygous mice harboring the targeted *Lmna* mutation, *Lmna*^tm1Stw^, were previously shown to have progressive muscular dystrophy and dilated cardiomyopathy^22–24^. We observed a similar disease progression from 1 week to 1 month. But unlike the former study, which showed a significant decrease in mouse weight in 2-week-old *Lmna*^−/−^ mice^15^, we did not identify a significant difference in growth between wild type (WT) and *Lmna*^−/−^ mice at 1 week and 2 weeks of age (Fig. 1a). However, by 1 month of age *Lmna*^−/−^ mice exhibited severe growth retardation and cardiac dysfunction (Fig. 1a,b). This cardiac dysfunction was accompanied by increased myocardial fibrosis in *Lmna*^−/−^ mice at 1 month of age (Fig. 1c). No appreciable differences in myocardial fibrosis were observed between *Lmna*^−/−^ and WT mice at either 1 week or 2 weeks of age.

### Early differentially expressed genes (DEGs) and related pathways in *Lmna*^−/−^ mice

**Figure 2:**
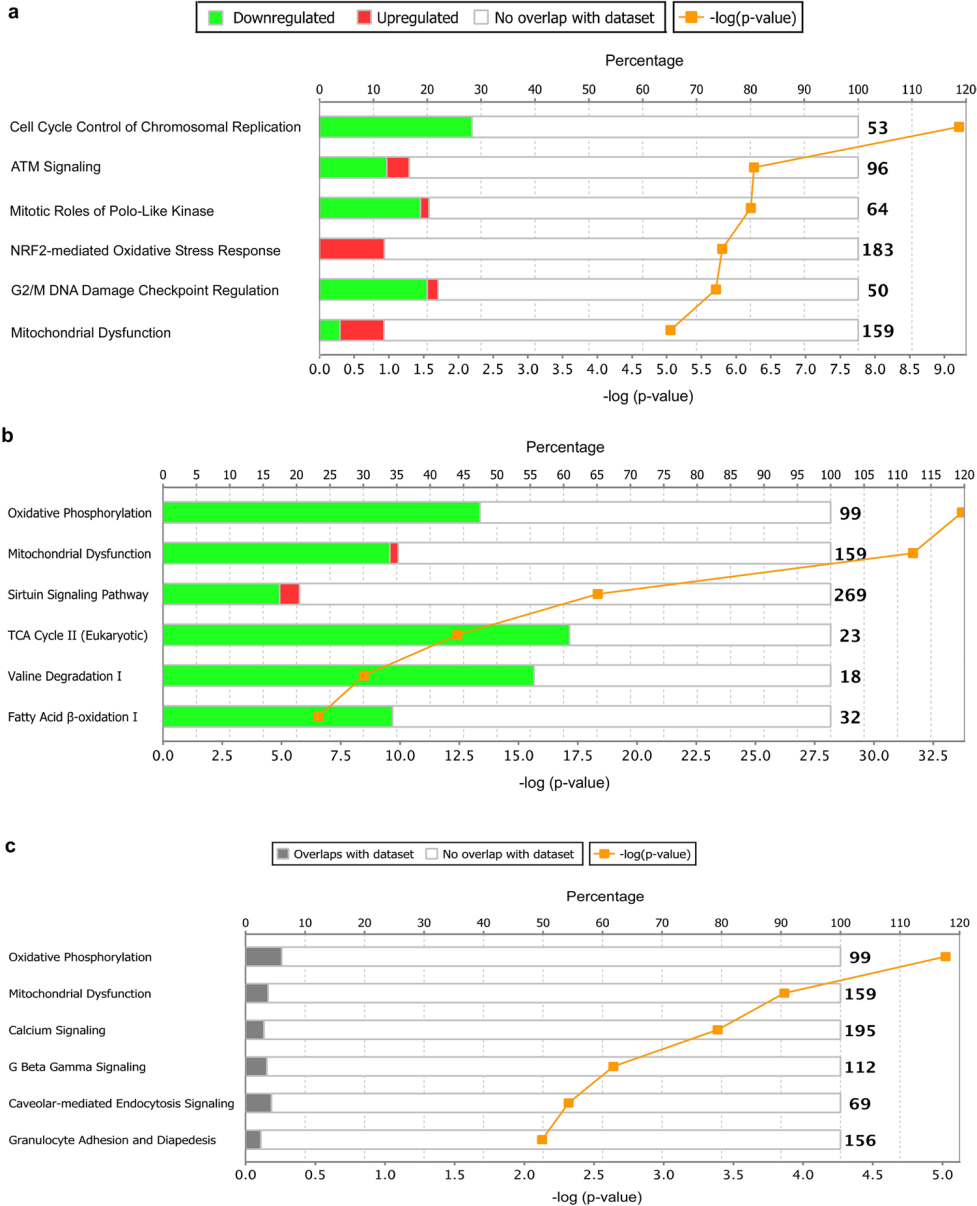
Top Canonical Pathways Based on the Differentially Expressed Genes (DEGs) Between WT and *Lmna*^−/−^ Mouse Hearts by Ingenuity Pathway Analysis (IPA). **(a)** Top 6 canonical pathways based on the DEGs between 2-week-old WT and *Lmna*^−/−^ mouse hearts; **(b)** Top 6 canonical pathways based on the DEGs between 1-month-old WT and *Lmna*^−/−^ mouse hearts; **(c)** Top 6 canonical pathways based on the overlapped DEGs between 2-week-old and 1-month-old *Lmna*^−/−^ mice. The bar graph shows the percentages of differentially expressed (both down- and up-regulated) genes among the genes in the IPA random dataset involved in the listed pathways. The number listed at the end of each pathway bar represents the number of all the genes in the IPA random dataset for that pathway. The line graph shows the significant differences between the DEGs (in 2-week-old *Lmna*^−/−^ vs. WT mouse hearts) and the genes in the IPA random dataset involved in the listed pathways. The point in each pathway line indicates the value of −log_10_ (p-value) for that specific pathway (between 5–9.25 in Fig. a, between 6–34 in Fig. b, between 2–5.5 in Fig. c). This transformation was performed because the p-values were very low (between 10^−5^–10^–9.25^ in Fig. a, between 10^−6^–10^−34^ in Fig. b, between 10^−2^–10^−5.5^ in Fig. c).

We first compared the gene expression profiles of WT and *Lmna*^−/−^ mice at 1 week and 2 weeks of age. Although *Lmna* was the only gene with significantly reduced expression in 1-week-old *Lmna*^−/−^ mice, we identified 730 DEGs (428 genes up-regulated, 293 genes down-regulated, 9 genes unmapped) in 2-week-old *Lmna*^−/−^ mice. Based on these genes, 58 related canonical pathways (with p < 0.05) were identified through IPA pathway analysis. Figure 2a and Supplementary Table S1 list the six pathways with the highest p values. Table 1 lists representative key DEGs in the related pathways/functions among 2-week *Lmna*^−/−^ mice. As shown in Table 1, *Lmna*^−/−^ mice at 2 weeks of age demonstrated both down- and up-regulation of the key genes involving cell cycle regulation (e.g. chromosomal replication down-regulated, counteractive effects on G1/S and G2/M transition, M phase progression repressed), mitochondrial dysfunction, oxidative phosphorylation, and apoptosis. Meanwhile, *Lmna*^−/−^ mice showed down-regulation in key genes in DNA damage response/repair pathways as well as up-regulation in genes involved in the oxidative stress response, acute phase response, cell survival/growth, cardiac hypertrophy, glycolysis, and triglyceride hydrolysis. We identified the up-regulation of *FOXO3* (listed in Table 1 apoptosis pathway), which was also found in a previous study in 2-week *Lmna*^−/−^ mice by Marian *et al*.^15^. However, instead of activation of the pathway in 2-week *Lmna*^*D300N*^ mice shown by Marian *et al*.^16^, we identified overall down-regulation of DNA damage response pathway (z score = −1.265). But we did observe the overall up-regulation of TP53 pathway (z score = 0.302) due to up-regulation of TP53 effector (*PERP*) and other TP53 target genes, which was again similar to the findings from Marian *et al*. in 2-week *Lmna*^*D300N*^ mice^16^.

**Table 1:** Examples of key DEGs in related pathways/functions among 2-week *Lmna*^−/−^ mice.

Pathways/Functions	Gene name	Gene symbol	Up(↑)- or down(↓)-regulated
**Cell cycle regulation:**			
Chromosomal replication ↓	Cell division cycle 6/7	*CDC 6/7*	↓
	Cyclin dependent kinase 1	*CDK1*	↓
	Minichromosome maintenance complex component 2–7	*MCM 2–7*	↓
	DNA polymerase epsilon	*POLE*	↓
G1/S transition ↓	Cyclin E2	*CCNE2*	↓
G1/S transition ↑	RB transcriptional corepressor 1	*RBL1*	↓
	Checkpoint kinase 1	*CHK1*	↓
G2/M transition ↓	Cyclin dependent kinase inhibitor 1 A	*CDKN1A*	↑
	CDC28 protein kinase regulatory subunit 2	*CKS2*	↓
	Cyclin dependent kinase 1	*CDK1*	↓
	Cyclin B	*CCNB*	↓
G2/M transition ↑	Checkpoint kinase 1	*CHK1*	↓
M phase progression ↓	Polo Like Kinase 1	*PLK1*	↓
	Cyclin dependent kinase 1	*CDK1*	↓
	Cyclin B	*CCNB*	↓
	Protein regulator of cytokinesis 1	*PRC1*	↓
	Kinesin family member 11/23	*KIF11/23*	↓
**Mitochondrial dysfunction/Oxidative phosphorylation**	Genes for enzymes in Complex I		↑
	Genes for enzymes in Complex III, IV, V		↓
**Apoptosis**	Forkhead box O3	*FOXO3*	↑
	BCL2 like 11	*BCL2L11*	↑
	BCL2 interacting protein 3	*BNIP3*	↑
	NF-kappa-B inhibitor alpha	*NFKBIA*	↑
	TP53 apoptosis effector	*PERP*	↑
	Period circadian regulator 1	*PER1*	↑
	Protein phosphatase 1 regulatory subunit 15 A	*PPP1R15A*	↑
	Baculoviral IAP repeat containing 5	*BIRC5*	↓
**DNA damage response/Repair ↓**	Breast cancer type 1 susceptibility protein	*BRCA1*	↓
	BRCA1 associated RING domain 1	*BARD1*	↓
	Bloom syndrome RecQ like helicase	*BLM*	↓
	BRCA1 interacting protein C-terminal helicase 1	*BRIP1*	↓
	Breast cancer type 2 susceptibility protein	*BRCA2*	↓
	Fanconi anemia complementation group D2	*FANCD2*	↓
	Alpha thalassemia/mental retardation syndrome X-linked chromatin remodeler	*ATRX*	↓
	High mobility group box 2	*HMGB2*	↓
**Oxidative stress response ↑**	Nuclear factor erythroid 2 like 2	*NRF2*	↑
	Actin gamma 1	*ACTG1*	↑
	Jun pro-oncogene, AP-1 transcription factor Subunit	*JUN*	↑
	Sequestosome 1	*SQSTM1*	↑
	Ferritin light chain	*FTL*	↑
	Four and a half LIM domains 1	*FHL1*	↑
	Epoxide hydrolase 1	*EPHX1*	↑
	Glutathione S-transferase mu 5	*GSTM5*	↑
**Acute phase response ↑**	Angiotensinogen	*AGT*	↑
	Protein kinase B beta	*AKT2*	↑
	Complement component 1 subcomponent R	*C1R*	↑
	Complement Component 3 and 4	*C3/4*	↑
	Nuclear factor of interleukin 6	*NFIL6*	↑
	Ceruloplasmin	*Cp*	↑
	Serpin family A member 3	*SERPINA3*	↑
	Serpin family G member 1	*SERPING1*	↑
	Fos proto-oncogene, AP-1 transcription factor subunit	*FOS*	↑
	Jun pro-oncogene, AP-1 transcription factor Subunit	*JUN*	↑
	NF-kappa-B inhibitor alpha	*NFKBIA*	↑
**Cell survival/growth**	Protein kinase B beta	*AKT2*	↑
	Pyruvate dehydrogenase kinase 2	*PDK2*	↑
	BCL2 like 1	*BCL2L1*	↑
	Insulin receptor substrate 2	*IRS2*	↑
	Insulin like growth factor binding protein 3/5/6	*IGFBP3/5/6*	↑
	Fos proto-oncogene, AP-1 transcription factor subunit	*FOS*	↑
**Cardiac hypertrophy**	Adrenoceptor alpha 1 A	*ADRA1A*	↑
	G protein subunit α	*GNA*	↑
	Heart and neural crest derivatives expressed 2	*HAND2*	↑
	Jun pro-oncogene, AP-1 transcription factor Subunit	*JUN*	↑
	Mitogen-activated protein kinase kinase kinase 6	*MAP3K6*	↑
	Mitogen-activated protein kinase-activated protein kinase 2	*MAPKAPK2*	↑
	Ras homolog family member B/J	*RHOB/J*	↑
**Glycolysis**	Fructose-bisphosphate A aldolase	*ALDOA*	↑
	Glucose-6-phosphate isomerase	*GPI*	↑
	Phosphofructokinase	*PFK*	↑
	Triosephosphate isomerase 1	*TPI1*	↑
**Triglyceride hydrolysis**	Patatin like phospholipase domain-containing protein 2	*PNPLA2*	↑

Taken together, 2-week-old *Lmna*^−/−^ mice demonstrated the activation of cell cycle arrest and apoptosis pathways and a failure of DNA repair in response to the targeted *Lmna* knockout mutation. Although the trend of cell cycle arrest and apoptosis seemed clear, some counteractive activation pathways were seen as well. For example, multiple cell survival factors were up-regulated to keep cells growing. In addition, while mitochondrial dysfunction (down-regulated genes for Complex III, IV, V) might compromise ATP generation and cause oxidative stress, other enzyme-coding genes for Complex I, III, IV, V and glycolysis were up-regulated to overcome ATP shortage, and the NRF2-mediated oxidative stress response pathway was dramatically activated to reduce oxidative damage. These counteractive effects might explain the lack of observed growth retardation at 2 weeks. In the meantime, up-regulation of the genes in cardiac hypertrophy, myofibroblast activation/fibrosis and acute phase response signaling might contribute to the early pathological changes before *LMNA* cardiomyopathy occurs.

### Mitochondrial dysfunction and diminished nutrient metabolism associated with extensive late-stage DEGs in 1-month-old *Lmna*^−/−^ mice

We identified 1004 DEGs between the gene expression profiles of WT and *Lmna*^−/−^ mice at 1 month of age, with 699 genes had decreased expression levels while 290 genes had increased expression levels (15 genes unmapped) in *Lmna*^−/−^ mice. Pathway analysis via IPA identified 66 canonical pathways (p < 0.05) based on these genes. Figure 2b and supplementary Table S2 list the top six pathways. Table 2 lists the examples of key DEGs in the related pathways/functions among 1-month-old *Lmna*^−/−^ mice. Most impressively, these mice demonstrated extensive down-regulation of key genes associated with mitochondrial dysfunction, oxidative phosphorylation, a wide range of metabolic pathways (tricarboxylic acid cycle, glycolysis, glycogen synthesis, glycogenolysis, amino acid degradation, fatty acid β-oxidation, ketogenesis, and ketolysis), cardiac β-adrenergic signaling, G protein β γ signaling, caveolar-mediated endocytosis, and cardiac voltage-gated channels (Table 2). Such alterations in pathways of metabolism and energy status in *Lmna*^−/−^ mice may explain their remarkable growth retardation. Down-regulation of multiple voltage-gated channels that have been reported to be related to the generation of arrhythmias^25^ - *KCNA5, KCND2, KCNE1, SCN4A, SCN4B* (listed in Table 2) - might be the underlying basis for the increased risk of arrhythmogenic events in *LMNA* cardiomyopathy. Meanwhile, we observed down- and up-regulated genes among these mice involved in cell survival signaling, cardiac hypertrophy, calcium signaling and autophagy (Table 2).

**Table 2:** Examples of key DEGs in related pathways/functions among 1-month *Lmna*^−/−^ mice.

Pathways/Functions	Gene name	Gene symbol	Up (↑)-/down (↓)-regulated
Mitochondrial dysfunction	Peroxiredoxin 3/5	*PRDX 3/5*	↑
	Superoxide dismutase 2	*SOD2*	↑
	Thioredoxin 2	*TXN2*	↑
	Apoptosis inducing factor mitochondria associated 1	*AIFM1*	↑
	Cytochrome C1	*CYC1*	↑
Oxidative phosphorylation	Genes for enzymes in all complex		↓
Cardiac β-adrenergic signaling	G protein subunit gamma	*GNG*	↑
	cAMP-dependent protein kinase inhibitor alpha	*PKIA*	↑
	Sarcoplasmic/endoplasmic reticulum Ca2+ ATPase 2	*ATP2A2*	↑
	Ryanodine receptor 2	*RYR2*	↑
G protein β γ signaling	G protein subunit gamma	*GNG*	↑
	G protein-coupled inwardly-rectifying potassium channel	*GIRK*	↑
Caveolar-mediated endocytosis	Caveolin 1	*CAV1*	↑
	Filamin B	*FLNB*	↑
	Integrin subunit alpha 1/6/8/9	*ITGA1/6/8/9*	↑
	Integrin subunit beta 6	*ITGB6*	↑
Voltage-gated channels (Action Potential generation)	Potassium voltage-gated channel subfamily A member 5	*KCNA5*	↑
	Potassium voltage-gated channel subfamily D member 2	*KCND2*	↑
	Potassium voltage-gated channel subfamily E regulatory subunit 1B	*KCNE1B*	↑
	Potassium voltage-gated channel modifier subfamily V member 2	*KCNV2*	↑
	Sodium voltage-gated channel alpha subunit 4	*SCN4A*	↑
	Sodium voltage-gated channel beta subunit 4	*SCN4B*	↑
Cell survival signaling	G protein subunit gamma	*GNG*	↓
	G protein-coupled inwardly-rectifying potassium channel	*GIRK*	↓
	Vascular endothelial growth factor A/B	*VEGFA/B*	↓
	Regulated protein tyrosine phosphatase receptor type F	*PTPRF*	↑
Cardiac hypertrophy	Calcium voltage-gated channel subunit alpha1G/H	*CACNA1G/H*	↑
	Transforming growth factor beta	*TGFβ*	↑
	Insulin like growth factor 1 receptor	*IGF1R*	↑
	Histone deacetylase 6	*HDAC6*	↑
	Protein phosphatase 3 catalytic subunit beta	*PPP3CB*	↓
Calcium signaling	Ryanodine receptor 2	*RYR2*	↓
	Troponin I3	*TNNI3*	↓
	Tropomyosin 4	*TPM4*	↓
	Troponin T2	*TNNT2*	↑
	Calcium/calmodulin dependent protein kinase 1G	*CAMK1G*	↑
	Calcium/calmodulin dependent protein kinase kinase 2	*CAMKK2*	↑
Autophagy	Autophagy related 4D cysteine peptidase	*ATG4D*	↓
	Autophagy related 3	*ATG3*	↑

Several up-regulated genes in 1-month *Lmna*^−/−^ mice are associated with sarcomere structure and ERK1/2 pathway. Compared with previous microarray studies by Worman *et al*. on mouse *LMNA* cardiomyopathy from *Lmna*^*H222P/H222P*^ mutation^7,26^, we identified up-regulation of a novel gene associated with sarcomere structure, myomesin 2 (*MYOM2*). *MYOM2* is a protein coding gene for M-band in sarcomere that plays an important role in maintaining sarcomere structure^27,28^. In addition, we identified up-regulation of dual specificity phosphatase 4 (*DUSP4*) and *DUSP5* in 1-month *Lmna*^−/−^ mice, while only *DUSP4* was up-regulated in mice with *Lmna*^*H222P/H222P*^-induced cardiomyopathy based on the study by Worman *et al*.^17^. DUSP4 and DUSP5 can be transcriptionally induced by ERK1/2, which suggests the possibility of ERK1/2 pathway activation in LMNA cardiomyopathy progression. In addition, we found some compensatory up-regulated genes in *Lmna*^−/−^ mice, e.g. AMP-Activated Protein Kinase Subunit Gamma-2 (*PRKAG2*) was up-regulated by higher cellular AMP/ATP ratio to inhibit key enzymes of ATP consuming pathways and induces ATP generation, and cAMP-dependent protein kinase type I alpha regulatory subunit (PRKAR1A) was up-regulated by cAMP accumulation for ATP generation and cell survival.

### Comparison of 2-week and 1-month differentially expressed genes among *Lmna*^−/−^ mice

While comparing the DEGs between 2-week-old and 1-month-old *Lmna*^−/−^ mice, we found more extensive gene expression changes at 1 month than at 2 weeks (1004 vs. 730), and DEGs in 1-month-old *Lmna*^−/−^ mice were mostly down-regulated while those in 2-week-old *Lmna*^−/−^ mice were mostly up-regulated. In addition, we found 96 DEGs that overlapped at both ages. These DEGs could be important because their expression changes start early (between 1–2 weeks of age), before an identifiable phenotype, and remain into the later life stage of *Lmna*^−/−^ mice, suggesting they may be related to the pathogenesis and progression of LMNA cardiomyopathy. Among the 96 overlapping DEGs, 64 genes are regulated in the same direction (either both up-regulated or both down-regulated) at the two time points while 32 genes are regulated in opposite directions (30 genes up-regulated at 2 weeks but down-regulated at 1 month and 2 genes down-regulated at 2 weeks but up-regulated at 1 month). Pathway analysis identified 23 canonical pathways (p < 0.05) based on the overlapping DEGs. Figure 2c and supplementary Table S3 listed the top six canonical pathways. Table 3 lists key overlapping DEGs, which were involved in oxidative phosphorylation, mitochondrial function, calcium signaling, G protein β γ signaling, and caveolar-mediated endocytosis. Although expression changes of some listed key genes could cause functional impairment at both time points, compensatory expression changes of other genes could prevent the occurrence of actual functional impairment as in 2-week-old *Lmna*^−/−^ mice. For example, the coding gene for Complex V enzyme MT-ATP6 was down-regulated in these mice (Table 3), which could affect ATP generation in mitochondria. However, the coding genes for Complex I/IV enzymes NDUFA13, NDUFS7, NDUFV1 and COX6A2 are simultaneously up-regulated (Table 3), producing more substrates for MT-ATP6 and protecting the mice from ATP shortage.

**Table 3:** Examples of key overlapped DEGs in related pathways/functions.

Pathways/Functions	Gene name	Gene symbol	At 2 weeks	At 1 month
Oxidative phosphorylation/Mitochondrial function	Cytochrome c oxidase subunit 6A2	*COX 6A2*	↑ (up-regulated)	↓ (down-regulated)
	NADH: ubiquinone oxidoreductase subunit A13	*NDUFA13*	↑	↓
	NADH: ubiquinone oxidoreductase core subunit S7	*NDUFS7*	↑	↓
	NADH: ubiquinone oxidoreductase core subunit V1	*NDUFV1*	↑	↓
	Mitochondrial ATP synthase subunit 6	*MT-ATP6*	↓	↓
Calcium signaling	Calcium voltage-gated channel subunit alpha 1 H	*CACNA1H*	↑	↑
	Troponin T2	*TNNT2*	↑	↑
	Myosin heavy chain 7	*MYH7*	↑	↑
	A-kinase anchoring protein 5	*AKAP5*	↓	↓
	Tropomyosin 4	*TPM4*	↓	↓
	Troponin I3	*TNNI3*	↑	↓
G protein β γ signaling	G protein subunit gamma 11	*GNG11*	↓	↓
	Caveolin 1	*CAV1*	↓	↓
	G protein subunit alpha o1	*GNAO1*	↑	↑
Caveolar-mediated endocytosis	Caveolin 1	*CAV1*	↓	↓
	Integrin subunit alpha 1	*ITGA1*	↓	↓
	Integrin subunit beta 5	*ITGB5*	↑	↑

Other important overlapping DEGs include those involved in glycogen degradation (acid α-glucosidase, phosphofructokinase - *PFKM*), glycolysis (*PFKM*), and lactose degradation (*prosaposin*). All these were up-regulated in 2-week-old and down-regulated in 1-month-old mice, suggesting more glucose generation and usage in 2-week-old *Lmna*^−/−^ mice and a dysfunction of glucose metabolism in 1-month-old mice. In addition, heat shock protein family B member 1 (*HSPB1*), nicotinamide riboside kinase (*NMRK2*) and dual specificity phosphatase 5 (*DUSP5*) were up-regulated at both time points. *HSPB1* is important for stress resistance and actin organization while *NMRK2* codes a key enzyme for NAD^+^ synthesis from nicotinamide riboside (NAD^+^ is one of the most important co-enzymes for redox reactions), and DUSP5, as a member of the dual specificity protein phosphatase subfamily, negatively regulates members of the mitogen-activated protein kinase (MAPK) superfamily (ERK1/2, stress-activated protein kinase/c-Jun N-terminal kinase - SAPK/JNK). *DUSP1* and *DUSP5* were up-regulated in 2-week-old *Lmna*^−/−^ mice while *DUSP4* and *DUSP5* were up-regulated in 1-month-old *Lmna*^−/−^ mice.

**Figure 3:**
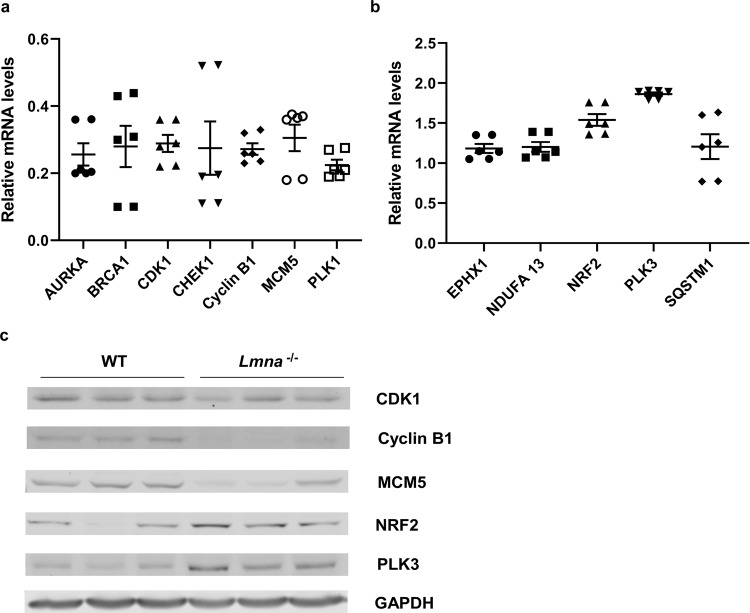
Validation of Two-week-old Mouse Heart RNA-Sequencing Results by RT-qPCR and Western Blot. RNA and protein samples analyzed were extracted from WT and *Lmna*^−/−^ mouse heart tissues at 2 weeks of age. (**a**) RT-qPCR validation of down-regulated genes (*AURKA, BRCA1, CDK1, CHEK1, Cyclin B1, MCM5, PLK1*) identified by RNA-Sequencing. The housekeeping gene S18 was used as a control gene for both WT and *Lmna*^−/−^ samples. ∆∆ cycle threshold (Ct) method was used for RT-qPCR analysis. Average WT expression level was normalized to one and *Lmna*^−/−^ expression levels were calculated over average WT level and showed as “mean ± SEM” on Y axis. The expression levels of all the listed genes were significantly lower in 2-week *Lmna*^−/−^ mouse hearts than in 2-week WT mouse hearts (p < 0.01). (**b**) RT-qPCR validation of up-regulated genes (*EPHX1, NDUFA13, NRF2, PLK3, SQSTM1*) identified by RNA-Sequencing. The same ∆∆Ct method was used for analysis. The relative expression levels of all the listed genes are showed as “mean ± SEM” on Y axis. The expression levels in 2-week-old *Lmna*^−/−^ mouse hearts were significantly higher than those in 2-week-old WT mouse hearts for all the genes (p < 0.05). (**c**) Western blot validation of protein expressions of representative down-regulated (*CDK1, Cyclin B1* and *MCM5*) and up-regulated (*NRF2* and *PLK3*) genes. GAPDH was used as a loading control. Three repeated experiments were conducted for each protein with similar results showed in the figure.

**Figure 4:**
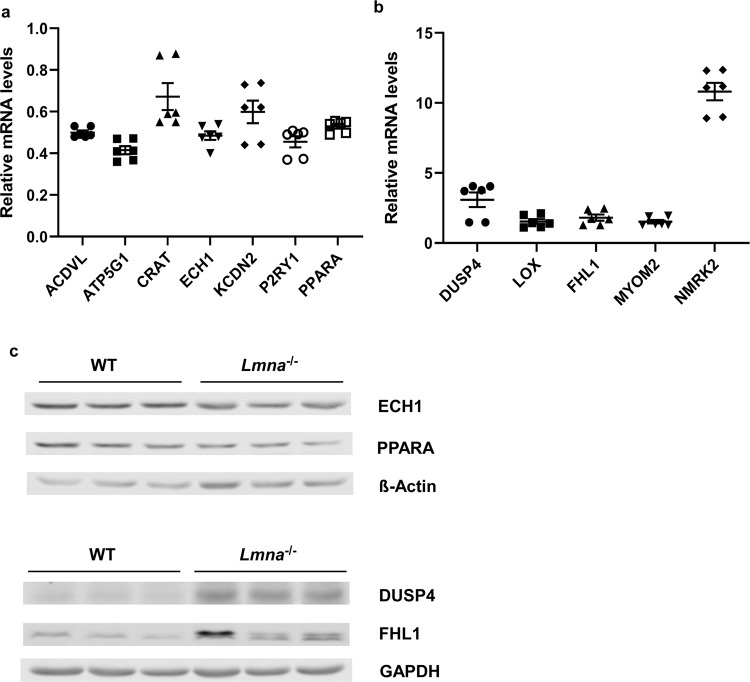
Validation of One-month-old Mouse Heart RNA-Sequencing Results by qRT-PCR and Western Blot. Samples (RNAs and proteins) being analyzed were extracted from WT and *Lmna*^−/−^ mouse heart tissues at 1 month of age. (**a**) RT-qPCR validation of down-regulated genes (*ACDVL, ATP5G1, CRAT, ECH1, KCDN2, P2RY1, PPARA*) identified by RNA-Sequencing. The same ∆∆Ct method was used for analysis as described in Fig. 3 legends. The relative expression levels of all the listed genes are showed as “mean ± SEM” on Y axis. The expression levels of all the listed genes among 1-month *Lmna*^−/−^ mouse hearts were significantly lower than the levels among 1-month WT mouse hearts (p < 0.05). (**b**) RT-qPCR validation of up-regulated genes (*DUSP4, LOX, FHL1, MYOM2, NMRK2*) identified by RNA-Sequencing. The same ∆∆Ct method was used for analysis. The relative expression levels of all the listed genes are showed as “mean ± SEM” on Y axis. The expression levels in 1-month *Lmna*^−/−^ mouse hearts were significantly higher than those in 1-month WT mouse hearts for all the genes (p < 0.05). (**c**) Western blot validation of protein expressions of representative down-regulated (*ECH1* and *PPARA*) and up-regulated (*DUSP4* and *FHL1*) genes. β-actin (for *ECH1* and *PPARA*) or GAPDH (for *DUSP4* and *FHL1*) was used as a loading control. Three repeated experiments were conducted for each protein with similar results showed in the figure.

To validate our RNA-sequencing results, we performed RT-qPCR and western blot for representative genes associated with the top 6 pathways (for 2 weeks, listed in Fig. 2a) as well as oxidation phosphorylation, mitochondrial function, fatty acid metabolism, sarcomere structure, and cardiac development for 1 month. RT-qPCR analysis confirmed decreased expression of Aurora Kinase A (*AURKA*), breast cancer type 1 susceptibility protein (*BRCA1*), cyclin dependent kinase 1 (*CDK1*), checkpoint kinase 1 (*CHEK1*), cyclin B1, minichromosome maintenance complex component 5 (*MCM5*), polo like kinase 1 (*PLK1*) (for 2 weeks, Fig. 3a), peroxisome proliferator activated receptor alpha (*PPARA*), carnitine o-acetyltransferase (*CRAT*), ATP synthase membrane subunit c locus1 (*ATP5G1*), enoyl-CoA hydratase 1 (*ECH1*), acyl-CoA dehydrogenase very long chain (*ACDVL*), purinergic receptor P2Y1 (*P2RY1*), potassium voltage-gated channel subfamily D member 2 (*KCDN2*) (for 1 month, Fig. 4a) and increased expression of epoxide hydrolase 1 (*EPHX1*), NADH: ubiquinone oxidoreductase subunit A13 (*NDUFA13*), nuclear factor, erythroid 2 like 2 (*NRF2*), polo like kinase 1 (*PLK3*), sequestosome 1 (*SQSTM1*) (for 2 weeks, Fig. 3b), dual specificity phosphatase 4 (*DUSP4*), lysyl oxidase (*LOX)*, ferritin heavy chain 1 (*FHL1*), myomesin 2 (*MYOM2*) and nicotinamide riboside kinase (*NMRK2*) (for 1 month, Fig. 4b) with statistically significant expression fold change (p < 0.05). Western blot analysis showed decreased protein levels of CDK1, cyclin B1, MCM5 (Fig. 3c), ECH1, PPARA (Fig. 4c) and increased protein levels of NRF2, PLK3 (Fig. 3c), DUSP4, FHL1 (Fig. 4c) in hearts from 2-week (Figs. 3c) and 1-month (Fig. 4c) *Lmna*^−/−^ mice compared to WT mice. The full-length western blot images are provided in Supplementary Fig. S1 (for Fig. 3c) and S2 (for Fig. 4c).

## Discussion

Our study is the first to utilize RNA sequencing and pathway analysis to set up detailed gene expression and pathway profiles over the course of *Lmna*^−/−^ induced *LMNA* cardiomyopathy, which may lead to further mechanistic studies of the pathogenesis and progression of *LMNA* cardiomyopathy. Based on an average lifespan of 5 weeks for *Lmna*^−/−^ mice, we selected three different time points that correspond to the early (1 week), middle (2 weeks), and late (1 month) stages of the disease to investigate the detailed molecular mechanisms over time in hearts isolated from WT and *Lmna*^−/−^ mice. The significantly increased number of genes with expression changes between 1 and 2 weeks (from 1 gene to 730 genes) and between 2 weeks to 1 month of age (from 730 genes to 1004 genes) was associated with the phenotypic observation with significant cardiac dysfunction and fibrosis.

The major findings of this study are the gene expression and related pathophysiological changes at different stages of *LMNA* cardiomyopathy. We have further identified how these changes evolved over time with disease progression. More importantly, we have identified 96 overlapping DEGs in 2-week-old and 1-month-old *Lmna*^−/−^ mice, which are mainly involved in oxidative phosphorylation, mitochondrial dysfunction, calcium signaling, and G protein beta gamma signaling. These findings have provided insights on the pathogenesis of *LMNA* cardiomyopathy due to loss of *LMNA* function.

We found the DEGs in 2-week-old *Lmna*^−/−^ mice were mainly involved in cell cycle regulation, DNA damage response, mitochondrial function, oxidative phosphorylation, oxidative stress response, and counteractive apoptosis/survival signaling. Although these mice seemed to fail in repairing their DNA damage, i.e. *Lmna* mutation (all related DEGs down-regulated) with down-regulated chromosomal replication and a trend of delayed cell cycle progression, we still observed some down-regulated genes meant to promote cell cycle progression. Similarly, up-regulated gene expression for mitochondrial complex I, III, IV and V enzymes might offset the consequences caused by down-regulated genes for complex III, IV, V enzymes for ATP synthesis from mitochondria. While oxidative stress may exist because of impaired oxidative phosphorylation, the oxidative stress response pathway was activated (all related DEGs up-regulated) to reduce oxidative injury. A similar pattern was observed with apoptosis and survival factors. There were up-regulated genes on both sides. These compensatory mechanisms might explain why the 2-week-old mice didn’t show any signs of *LMNA* cardiomyopathy or growth retardation yet.

For 1-month-old *Lmna*^−/−^ mice, we observed significant growth and cardiac phenotypes associated with extensive down-regulation of genes involving oxidation phosphorylation, mitochondrial function, almost all nutrient metabolism (e.g. TCA cycle, amino acid degradation, and fatty acid β-oxidation), cardiac β-adrenergic signaling, G protein beta gamma signaling. Overwhelming oxidative stress and ATP deficiency caused by seriously impaired oxidation phosphorylation and mitochondrial dysfunction, together with extensively reduced nutrient metabolism might explain the remarkable growth retardation of *Lmna*^−/−^ mice at this stage. Up-regulated cardiac β-adrenergic signaling (z score = 1) and dysregulated G protein beta gamma signaling (z score =0) in the heart could also contribute to cardiac dysfunction we observed. Our study provided a unique model that could identify comprehensive and accurate gene expression levels using RNA sequence analysis to further understand signaling pathways involved in the progressive dilated cardiomyopathy process from *Lmna* mutation.

Our study is the first to report that mitochondrial dysfunction and impaired oxidative phosphorylation can occur in *Lmna*^−/−^ mice as early as 2 weeks of age, even before an apparent phenotype of *LMNA* cardiomyopathy. At late disease stage (1 month of age), when the phenotypes are dramatic, these impairments are more prominent. 47.5% genes involving oxidative phosphorylation and 35.2% genes involving mitochondrial dysfunction are down-regulated. Mitochondrial dysfunction has been described in dilated or hypertrophic cardiomyopathies and heart failure but its exact mechanism is unclear^29–32^. Mitochondrial dysfunction further promotes increased reactive oxygen species^29,33^. Mitochondrial dysfunction and increased oxidative stress have been noted in progeria laminopathies^34–37^. A specific gene targeted therapy or drug therapy to reduce oxidative stress has shown improvement of progeria phenotype^34,35^. It was previously shown that there was increased oxidative stress in heart failure and efforts had been made to target the oxidative stress pathway to prevent heart failure progression^38,39^. Our study suggested the possibility that reduction of oxidative stress might prevent or delay the development from *Lmna* mutation to *LMNA* cardiomyopathy.

Increased reactive oxidative stress and mitochondrial dysfunction can also affect ERK1/2 as well as mTOR pathways^40,41^. The over-activation of ERK1/2 and mTOR pathways has been well described in *LMNA* cardiomyopathy^8,9,11,13^. Our RNA sequence analysis shows increased gene expression level of dual specificity phosphatase 1 - *DUSP1* (dephosphorylates and inactivates ERK2) and *DUSP5* (inactivates ERK1) at 2 weeks of age as well as up-regulated *DUSP4* (inactivates ERK1, ERK2 and JNK), *DUSP5* and down-regulated *DUSP7, DUSP8* (inactivates SAPK/JNK and p38) and *DUSP18*, suggesting over-activation of ERK1/2 signaling pathway. We also find that mitogen-activated protein kinase kinase kinase 6 - MAP3K6 (upstream kinase of MAPK pathways) and mitogen-activated protein kinase-activated protein kinase 2 - MAPKAPK2 (regulated through direct phosphorylation by p38 MAPK) are up-regulated in 2-week-old *Lmna*^−/−^ mice. Meanwhile, we observe multiple up-regulated genes involving insulin like growth factor 1 (IGF1) signaling including *Akt2* in 2-week-old *Lmna*^−/−^ mice, which may promote mTOR activation. In addition, we identify a novel coding gene for nicotinamide riboside kinase (NMRK2) that is significantly up-regulated in *Lmna*^−/−^ mice. NMRK2 has been shown to play a key role in myogenesis especially through its interactions with integrins thereby activating the integrin-mediated signaling pathways^42,43^. ERK1/2 pathway is a well-known downstream effector of integrin-mediated signaling pathway and our finding provides further insights of ERK1/2 signaling mechanisms in *LMNA* cardiomyopathy.

Besides identifying the importance of oxidative stress in the pathogenesis of *LMNA* cardiomyopathy, our study is the first to report the upregulation of counteractive factors and signaling pathways for both cell apoptosis and survival in 2-week *Lmna*^−/−^ mice before the appearance of disease phenotypes. The upregulated pro-apoptotic factors include forkhead box O3 (*FOXO3*), BCL2 like 11 (*BCL2L11, BIM*), BCL2 interacting protein 3 (*BNIP3*), TP53 apoptosis effector (*PERP*), *et al*. (Table 1). Some of these factors are targets of *FOXO3* and *TP53*, suggesting the upregulation of FOXO3 and TP53 pathways. Upregulated TP53 pathway (p = 0.00233, z score 0.302) is identified by IPA pathway analysis based on 2-week DEGs. The upregulated survival factors include protein kinase B beta (*Akt2*), pyruvate dehydrogenase kinase 2 (*PDK2*), BCL2 like 1 (*BCL2L1, BCL-XL*), insulin receptor substrate 2 (*IRS2*), insulin like growth factor binding protein 3/5/6 (*IGFBP3/5/6*), *et al*. (Table 1). We observed significantly upregulated IGF-1 pathway (p = 0.00545, z score 2.236), NRF2-mediated oxidative stress response (p = 1.59E-6, z score 3.207) and acute phase response signaling (p = 9.42E-4, z score 3.317), which may work together to promote cell survival and growth. It is reasonable to think these may be compensatory mechanisms under the background of DNA damage (deletion mutation), failure of DNA damage repair and counteractive factors on cell cycle progression in order to keep 2-week-old *Lmna*^−/−^ mice in temporarily similar overall growth as that of their WT littermates. Interventions which inhibit cell apoptosis or enhance survival pathways may delay the appearance of disease phenotypes and improve the lifespan of *Lmna*^−/−^ mice.

Activated FOXO1/3 and their target genes have been demonstrated in 2-week *Lmna*^−/−^ mice by Auguste G *et al*.^15^. They also found suppression of FOXO1/3 activation by shRNA down-regulated the target gene transcription, improved apoptosis and prolonged survival of 2-week mice by 2-fold^15^. Conformed to this study, our study also finds the up-regulation of *FOXO3* and its target genes (*BCL2 L11/ BIM, BCL6, BNIP3* for apoptosis/autophagy, *P21CIP1, PLK3*, down-regulated *PLK1/4* and cyclin B1/2 for cell cycle arrest). We don’t detect the up-regulation of the target genes for DNA repair and reactive oxygen species detoxification. Although *FOXO1* gene expression is not found up-regulated in our analysis, we do identify up-regulated expression of its target genes as well. No up-regulation of *FOXO4* and its target genes are found in 2-week *Lmna*^−/−^ mice. But we observe mostly down-regulated target genes for *FOXO 1/3/4* in 1-month *Lmna*^−/−^ mice although no gene expression level changes for these three FOXO factors.

FOXO3/1 proteins can be activated by DNA damage or oxidative stress^44^. But when the intensity of these stimuli increases beyond a certain threshold, they activate pro-apoptotic genes and genes for cell cycle arrest. Meanwhile, Akt is a major upstream regulator of FOXO factors^44–46^. Our study shows up-regulation of Akt2 (phosphorylates and inactivates FOXO) through IGF1 pathway, suggesting over-activation of FOXO3/1. In addition, over-activation of ERK1/2 (phosphorylates and inactivates FOXO3) in these mice as discussed above supports the existence of over-activation of FOXO3^47^. Combined with downstream and upstream changes, our study provides some potential mechanisms on FOXO3/1 activation in 2-week *Lmna*^−/−^ mice.

The activation of E2F transcription factor/DNA damage response/TP53 pathway has been found in a 2-week *Lmna*^*D300N*^ mouse model^16^. In our study, E2F is predicted to be inhibited (p = 1.30E-8, z score −2.798) in 2-week *Lmna*^−/−^ mice because all of its 10 target genes (among DEGs) are inhibited. For DNA damage response, all the related DEGs in 2-week *Lmna*^−/−^ mice are down-regulated. However, our study shows TP53 is predicted to be activated (p = 5.88E-24, z score 3.977) with 46 target genes up-regulated in 2-week *Lmna*^−/−^ mice. The data differences between our study and the previous study may be due to the difference of mouse models and different lifespan: *Lmna*^*D300N*^ mice at 30 days vs*. Lmna*^−/−^ mice at 42 days. At 2 weeks, these two kinds of mice may be at different disease stages, and their data may not be comparable.

In summary, our study identified failure of DNA repair, dysregulated cell cycle progression and mitochondrial dysfunction/impaired oxidative phosphorylation associated with loss of *LMNA*, thereby providing mechanistic insights to *LMNA* cardiomyopathy and heart failure. Compared with previous gene profiling using microarray in *Lmna*^*H222P/H222P*^-induced cardiomyopathy (at 10 weeks of age)^7,26^ and RNA sequencing study in 2-week *Lmna*^−/−^ mice^15^ and *Lmna*^*D300N*^ mice^16^, our study not only confirmed the previously known genes and pathways related to *LMNA* cardiomyopathy but also identified novel genes and pathways that could contribute to the pathogenesis and progression of *LMNA* cardiomyopathy. It will be interesting to further investigate whether we can apply the same analysis to peripheral samples for its better practicality and feasibility. This application will provide us with detailed information at genomic level which can guide us to better understand this devastating disease in human subjects and develop advanced treatments including gene therapy.

## Methods

### Animals

Animal use and the study protocol were approved by the Institutional Animal Care and Use Committee at the Cleveland Clinic in accordance with the United States Public Health Service Policy on the Humane Care and Use of Animals, and the NIH Guide for the Care and Use of Laboratory Animals. Heterogeneous C57BL/6 mice harboring the targeted *Lmna* mutation, *Lmna*^tm1Stw^, were purchased from the Jackson Laboratory (stock# 009125). Heterozygotes and littermate wild type (WT) C57BL/6 mice were inbred and LMNA gene mutation was confirmed by genotyping^22,23,48^. 8 WT and 8 *Lmna*^−/−^ mice were used for each time point at 1 week, 2 weeks and 1 month of age. Hearts were isolated from WT and homozygous *Lmna*^−/−^ mice at each time point. Hearts were rinsed with cold saline to remove blood, snap frozen in liquid nitrogen, and stored at −80 °C for future use or fixed in 10% formalin for histology examination.

### Transthoracic echocardiography

Mice at 1 month of age were anesthetized with isoflurane inhalation and placed on a table. Echocardiography was performed using a GE Vivid 7 ultrasound with a 14 MHz transducer (GE Healthcare, Chicago, IL). Left ventricular shortening fraction was measured in 2D mode.

### Histological examination

Formalin-fixed, paraffin-embedded left ventricular (LV) sections were prepared and stained with Picrosirus Red. Five to six regions were randomly selected from each section and fibrosis areas were analyzed by an independent researcher, viewing in the whole heart longitudinal and cross sections with Image Pro Plus v7.0 software (Media Cybernetics Inc, Rockville, MD). Fibrosis area was presented as ratio of fibrotic areas to the whole myocardium areas^49^.

### Protein extraction and Western blot analysis

Each mouse heart was placed in an ice-cold lysis buffer containing 50 mM Tris-HCl (pH 7.4), 150 mM NaCl, 1 mM EDTA, 1% Tergitol solution NP-40, 25 mM β-glycerolphosphate, 10% glycerol, proteinase and phosphatase inhibitors, and homogenized immediately using an Omni TH tissue homogenizer (Omni International, Kennesaw, Georgia). Samples were then centrifuged at 2,000 rpm for 10 min at 4 °C and supernatant was stored at −80 °C for Western blot analysis. 50–60 µg of total proteins were resolved in SDS-PAGE, and transferred to nitrocellulose membranes. The membranes were blocked with 5% skim dry milk in 1x TBST for 1 hour and blotted with primary antibodies including anti-CDK1 (1:500, #77055, Cell Signaling), anti-Cyclin B1 (1:250, #4138, Cell Signaling), anti-MCM5 (1:1000, ab75975, Abcam), anti-NRF2 (1:250, #12721, Cell Signaling), anti-PLK3 (1:250, #4896, Cell Signaling), anti-ECH1 (1:500, PA5-30012, Thermo Fisher Scientific), anti-PPARA (1:500, ab126285, Abcam), anti-DUSP4 (1:100, sc-17821, Santa Cruz), anti-FHL1 (1:500, ab133661, Abcam), anti-GAPDH (1:2000, PA1-987, Thermo Fisher Scientific) and anti-β-actin (1:100, sc-81178, Santa Cruz) in 5% skim dry milk in 1x TBST at 4 °C overnight except anti-GAPDH was incubated at room temperature for 1 hour. LI-COR IRDye 800CW goat anti-mouse IgG (925–32210), goat anti-rabbit IgG (925–32211) or donkey anti-goat IgG (925–32214) were used as secondary antibodies at 1:5000 dilution for 1 hour at room temperature. The membranes were scanned using an Odyssey infrared imager (LI-COR, Lincoln, Nebraska)^50^.

### RNA isolation and RNA sequence analysis

The hearts were harvested at three time points: 1 week, 2 weeks and 1 month of age, as postnatal growth of the homozygous mice is still not retarded at one week old of age but is severely retarded at 1 month of age. The entire heart tissue was used for RNA extraction to avoid variance caused by sampling different part of the heart tissue. Three hearts from WT and *Lmna*^−/−^ at each time point (total 18 hearts) were used for this experiment. Total RNA was extracted and purified using RNeasy Fibrous Tissue Mini Kit (QIAGEN, Germantown, MD). RNA concentrations were measured using NanoDrop Spectrophotometers. Quality of the RNA samples was analyzed using the Agilent Bioanalyzer and all samples submitted for sequencing had a RNA Integrity number (RIN) > 8. Truseq Stranded Total RNA -RiboZero library preparation and RNA sequencing were performed by the Cleveland Clinic Lerner Research Institute Genomic Core. RNA sequencing was performed using the Illumina SBS v2 chemistry with 2 × 100 bp pair-end reads. The RNA-Sequencing data were aligned using STAR (v2.5.2b) program with default parameters^51^. Mapped read pairs were assigned to genes by collapsing all transcripts into a single gene model and then counting the number of reads that fully overlap the resulting exons. Reads that mapped to multiple locations were only counted once and those mapping to ambiguous regions were excluded. Reads uniquely mapped were considered for further analysis. DEseq. 2 (v3.8), a program using count-based matrices to identify differentially expressed genes (DEGs), was used to determine gene signatures^51^. To improve the reliability and accuracy of differential expression analysis, only genes with raw counts >5 in all individually sequenced samples were examined and compared between WT vs. *Lmna*^−/−^ at 1 week of age, at 2 weeks of age and at 1 month of age. For differentially expressed genes, INGENUITY Pathway Analyses (IPA, Qiagen, Hilden Germany) were performed, including enrichment analysis for canonical pathway, disease and biological function, toxicology function.

### Validation of the RNA-sequencing data

The most changed genes in the interesting signaling pathways were further confirmed using RT-qPCR and Western blot. RT-qPCR was performed using the TaqMan protocol in StepOnePlus Real-Time PCR System (Applied Biosystems, Foster City, CA). The TaqMan gene expression assays for the following genes were purchased from Applied Biosystems: *AURKA* (Mm01248177_m1), *BRCA1* (Mm00515386_m1), *CDK1* (Mm00772472_m1), *CHEK1* (Mm01176757_m1), *Cyclin B1* (Mm00838401_g1), *EPHX1* (Mm00468752_m1), *MCM5* (Mm01243769_m1), *NDUFA13* (Mm00445751_m1), *NRF2* (Mm00477784_m1), *PLK1* (Mm00440924_g1), *PLK3* (Mm00457348_m1), *SQSTM1* (Mm00448091_m1), *ECH1* (Mm00469322_m1), *PPARA* (Mm00440939_m1), *ATP5G1* (Mm02601566_g1), *ACADVL* (Mm00444293_m1), *CRAT* (Mm00483985_m1), *P2RY1* (Mm02619947_s1), *KCND2* (Mm01161732_m1), *LOX* (Mm00495386_m1), *FHL1* (Mm04204611_g1), *MYOM2* (Mm00500665_m1), *LMNA* (Mm00497783_m1), *DUSP4* (Mm00723761_m1), and *NMRK2* (Mm01172899_g1). The housekeeping gene *RPS18* (Mm02601777_g1) was used as control gene. Gene fold change calculation was determined by ∆∆Ct method over WT controls.

### Statistical analysis

Data are expressed as the mean ± standard deviation (SD). Graphpad (Prism Software) was used for statistical analysis. Statistical significance was determined by 2-tailed Student’s t-test with a value of p < 0.05 considered significant.

## Supplementary information

Supplementary information.

## Data Availability

The RNA sequencing data generated and analyzed during this study have been deposited in NCBI’s Gene Expression Omnibus (GEO) and are accessible through GEO series accession number GSE133693 (https://www.ncbi.nlm.nih.gov/geo/query/acc.cgi?acc=GSE133693) and token number ahqvgqmypbexjgn.

## References

[1] Hershberger RE, Siegfried JD (2011). Update 2011: clinical and genetic issues in familial dilated cardiomyopathy. J. Am. Coll. Cardiol..

[2] Prokocimer M (2009). Nuclear lamins: key regulators of nuclear structure and activities. J. Cell Mol. Med..

[3] Gruenbaum Y, Foisner R (2015). Lamins: nuclear intermediate filament proteins with fundamental functions in nuclear mechanics and genome regulation. Ann. Rev. Biochem..

[4] Shimi T (2008). The A- and B-type nuclear lamin networks: microdomains involved in chromatin organization and transcription. Genes Dev..

[5] Maraldi NM, Capanni C, Cenni V, Fini M, Lattanzi G (2011). Laminopathies and lamin-associated signaling pathways. J. Cell Biochem..

[6] Genetics Home Reference: LMNA gene. National Institutes of Health. National Library of Medicine, https://ghr.nlm.nih.gov/gene/LMNA (2020).

[7] Muchir A (2007). Activation of MAPK pathways links LMNA mutations to cardiomyopathy in Emery-Dreifuss muscular dystrophy. J. Clin. Invest..

[8] Muchir A, Wu W, Worman HJ (2010). Mitogen-activated protein kinase inhibitor regulation of heart function and fibrosis in cardiomyopathy caused by lamin A/C gene mutation. Trends Cardiovasc. Med..

[9] Wu W, Muchir A, Shan J, Bonne G, Worman HJ (2011). Mitogen-activated protein kinase inhibitors improve heart function and prevent fibrosis in cardiomyopathy caused by mutation in lamin A/C gene. Circulation..

[10] Choi JC (2012). Temsirolimus activates autophagy and ameliorates cardiomyopathy caused by lamin A/C gene mutation. Sci. Transl. Med..

[11] Muchir A (2012). Treatment with selumetinib preserves cardiac function and improves survival in cardiomyopathy caused by mutation in the lamin A/C gene. Cardiovasc. Res..

[12] Muchir A, Shan J, Bonne G, Lehnart SE, Worman HJ (2009). Inhibition of extracellular signal-regulated kinase signaling to prevent cardiomyopathy caused by mutation in the gene encoding A-type lamins. Hum. Mol. Genet..

[13] Ramos FJ (2012). Rapamycin reverses elevated mTORC1 signaling in lamin A/C-deficient mice, rescues cardiac and skeletal muscle function, and extends survival. Sci. Transl. Med..

[14] Wu W, Iwata S, Homma S, Worman HJ, Muchir A (2014). Depletion of extracellular signal-regulated kinase 1 in mice with cardiomyopathy caused by lamin A/C gene mutation partially prevents pathology before isoenzyme activation. Hum. Mol. Genet..

[15] Auguste G (2018). Suppression of Activated FOXO Transcription Factors in the Heart Prolongs Survival in a Mouse Model of Laminopathies. Circ. Res..

[16] Chen SN (2019). DNA Damage Response/TP53 Pathway Is Activated and Contributes to the Pathogenesis of Dilated Cardiomyopathy Associated With LMNA (Lamin A/C) Mutations. Circ. Res..

[17] Choi JC (2012). Dual specificity phosphatase 4 mediates cardiomyopathy caused by lamin A/C (LMNA) gene mutation. J. Biol. Chem..

[18] Muchir A (2013). Inhibition of extracellular signal-regulated kinase 1/2 signaling has beneficial effects on skeletal muscle in a mouse model of Emery-Dreifuss muscular dystrophy caused by lamin A/C gene mutation. Skelet. Muscle..

[19] Wang Z, Gerstein M, Snyder M (2009). RNA-Seq: a revolutionary tool for transcriptomics. Nat. Rev. Genet..

[20] Zhao S, Fung-Leung WP, Bittner A, Ngo K, Liu X (2014). Comparison of RNA-Seq and microarray in transcriptome profiling of activated T cells. PLoS One..

[21] Wang C (2014). The concordance between RNA-seq and microarray data depends on chemical treatment and transcript abundance. Nat. Biotechnol..

[22] Sullivan T (1999). Loss of A-type lamin expression compromises nuclear envelope integrity leading to muscular dystrophy. J. Cell Biol..

[23] Stewart CL, Kozlov S, Fong LG, Young SG (2007). Mouse models of the laminopathies. Exp. Cell Res..

[24] Frock RL (2006). Lamin A/C and emerin are critical for skeletal muscle satellite cell differentiation. Genes Dev..

[25] Dehghani-Samani A, Madreseh-Ghahfarokhi S, Dehghani-Samani A (2019). Mutations of Voltage-Gated Ionic Channels and Risk of Severe Cardiac Arrhythmias. Acta Cardiol. Sin..

[26] Muchir A (2012). Abnormal p38alpha mitogen-activated protein kinase signaling in dilated cardiomyopathy caused by lamin A/C gene mutation. Hum. Mol. Genet..

[27] Lange S (2005). Dimerisation of myomesin: implications for the structure of the sarcomeric M-band. J. Mol. Biol..

[28] van der Ven PF (1999). Assignment of the human gene for endosarcomeric cytoskeletal M-protein (MYOM2) to 8p23.3. Genomics..

[29] Arbustini E (1998). Mitochondrial DNA mutations and mitochondrial abnormalities in dilated cardiomyopathy. Am. J. Pathol..

[30] Marin-Garcia J, Goldenthal MJ, Ananthakrishnan R, Pierpont ME (2000). The complete sequence of mtDNA genes in idiopathic dilated cardiomyopathy shows novel missense and tRNA mutations. J. Card. Fail..

[31] Arbustini E (1998). Coexistence of mitochondrial DNA and beta myosin heavy chain mutations in hypertrophic cardiomyopathy with late congestive heart failure. Heart..

[32] Zhang D, Ezekiel UR, Chang SW, Zassenhaus HP (2005). Gene expression profile in dilated cardiomyopathy caused by elevated frequencies of mitochondrial DNA mutations in the mouse heart. Cardiovasc. Pathol..

[33] Raha S, Robinson BH (2000). Mitochondria, oxygen free radicals, disease and ageing. Trends in Biochemical Sciences..

[34] Kubben N (2016). Repression of the Antioxidant NRF2 Pathway in Premature. Aging. Cell..

[35] Rivera-Torres J (2013). Identification of mitochondrial dysfunction in Hutchinson-Gilford progeria syndrome through use of stable isotope labeling with amino acids in cell culture. J. Proteomics..

[36] Worman HJ, Fong LG, Muchir A, Young SG (2009). Laminopathies and the long strange trip from basic cell biology to therapy. J. Clin. Invest..

[37] Zaremba-Czogalla M, Dubinska-Magiera M, Rzepecki R (2011). Laminopathies: the molecular background of the disease and the prospects for its treatment. Cell Mol. Biol. Lett..

[38] Keith M (1998). Increased Oxidative Stress in Patients With Congestive Heart Failure 1. J. Am. Coll. Cardi..

[39] Tsutsui H, Kinugawa S, Matsushima S (2011). Oxidative stress and heart failure. Am. J. Physiol. Heart Circ. Physiol..

[40] Nacarelli T, Azar A, Sell C (2015). Aberrant mTOR activation in senescence and aging: A mitochondrial stress response?. Exp. Gerontol..

[41] Sieprath T, Darwiche R, De Vos WH (2012). Lamins as mediators of oxidative stress. Biochem. Biophys. Res. Commun..

[42] Li J, Mayne R, Wu C (1999). A novel muscle-specific beta 1 integrin binding protein (MIBP) that modulates myogenic differentiation. J. Cell Biol..

[43] Li J, Rao H, Burkin D, Kaufman SJ, Wu C (2003). The muscle integrin binding protein (MIBP) interacts with alpha7beta1 integrin and regulates cell adhesion and laminin matrix deposition. Dev. Biol..

[44] Carter ME, Brunet A (2007). FOXO transcription factors. Curr. Biol..

[45] Brunet A (1999). Akt promotes cell survival by phosphorylating and inhibiting a forkhead transcription factor. Cell..

[46] Kops GJPL (1999). Direct control of the forkhead transcription factor AFX by protein kinase B. Nature..

[47] Yang JY (2008). ERK promotes tumorigenesis by inhibiting FOXO3a via MDM2-mediated degradation. Nature cell biol..

[48] Frock RL (2012). Cardiomyocyte-specific expression of lamin improves cardiac function in Lmna−/− mice. PLoS One..

[49] Li W (2018). Paraoxonase 2 prevents the development of heart failure. Free Radic. Biol. Med..

[50] Shao Z (2012). Pulmonary Hypertension Associated with Advanced Systolic Heart Failure: Dysregulated Arginine Metabolism and Importance of Compensatory Dimethylarginine Dimethylaminohydrolase-1. J. Am. Coll. Cardiol..

[51] Love MI, Huber W, Anders S (2014). Moderated estimation of fold change and dispersion for RNA-seq data with DESeq. 2. Genome Biol..

