# Supplementary material for: RNA Sequence Analyses throughout the Course of Mouse Cardiac Laminopathy Identify Differentially Expressed Genes for Cell Cycle Control and Mitochondrial Function
Source: Sci Rep. 2020 Apr 20;10:6632. doi: 10.1038/s41598-020-63563-x (PMC7170950; doi:10.1038/s41598-020-63563-x)
Supplement: Supplementary file 1 — Supplementary information. [file 41598_2020_63563_MOESM1_ESM.pdf]

RNA Sequence Analyses throughout the Course of Mouse Cardiac Laminopathy Identify Differentially Expressed Genes for Cell Cycle Control and Mitochondrial Function

Zhili Shao, MD, PhD[a], Wonshill Koh, MD, PhD[b], Ying Ni, PhD[c], Wei Li, MD, PhD[d], Brendan Agatisa-Boyle[a], Daria Merkurjev, PhD[e], W. H. Wilson Tang, MD[a,f *]

[a]Department of Cardiovascular and Metabolic Sciences, Lerner Research Institute, Cleveland Clinic, Cleveland, OH; [b]Department of Cardiology, Cincinnati Children's Hospital Medical Center, Cincinnati, Ohio; [c]Department of Quantitative Health Sciences, Lerner Research Institute, Cleveland Clinic, Cleveland, OH; [d]Department of Biomedical Sciences, Joan C. Edwards School of Medicine, Marshall University, Huntington, WV; [e]Divison of Cardiology, Department of Medicine, University of California at Los Angeles, Los Angeles, CA; [f]Kaufman Center for Heart Failure, Department of Cardiovascular Medicine, Heart and Vascular Medicine, Cleveland Clinic, Cleveland, OH.

Supplementary Data (Tables S1-S3, Figures S1-S2)

Supplementary Table S1. Top canonical pathways that are associated with major differentially expressed genes (DEGs) in 2-week *Lmna*$^{-/-}$ mouse hearts.

Name	P value‡	Overlap	Z core
Cell Cycle Control of Chromosomal Replication	6.18E-10	15/53 (28.3%)	Not available
ATM* Signaling	5.51E-07	16/96 (16.7%)	-0.577
Mitotic Roles of Polo-Like Kinase	6.12E-07	13/64 (20.3%)	-1.897
NRF2†-mediated Oxidative Stress Response	1.59E-06	22/183 (12.0%)	3.207
G2/M DNA Damage Checkpoint Regulation	1.95E-06	11/50 (22%)	1.897
Mitochondrial Dysfunction	8.83E-06	19/159 (11.9%)	Not available

* Ataxia-telangiectasia mutated serine/threonine kinase

† = NFE2L2, Nuclear factor (erythroid-derived 2)-like 2

‡ P values were generated when the DEGs involving a specific pathway in 2-week *Lmna*$^{-/-}$ mice were compared with the genes involving the same pathway in the IPA random dataset.

Supplementary Table S2. Top canonical pathways that are associated with major differentially expressed genes (DEGs) in 1-month *Lmna*$^{-/-}$ mouse hearts.

Name	P value†	Overlap	Z score
Oxidative Phosphorylation	9.6E-34	47/99 (47.5%)	-6.856
Mitochondrial Dysfunction	2.24E-32	56/159 (35.2%)	Not available
Sirtuin Signaling Pathway	4.51E-19	55/269 (20.4%)	2.343
TCA* Cycle II (Eukaryotic)	3.8E-13	14/23 (60.9%)	-3.742
Valine Degradation I	3.36E-09	10/18 (55.6%)	-3.162
Fatty Acid β-oxidation I	2.7E-07	11/32(34.4%)	-3.317

* Tricarboxylic acid

† P values were generated when the DEGs involving a specific pathway in 1-month *Lmna*$^{-/-}$ mice were compared with the genes involving the same pathway in the IPA random dataset.

Supplementary Table S3. Top canonical pathways that are associated with overlapped DEGs of 2-week-old and 1-month-old *Lmna*$^{-/-}$ mice.

Name	P value*	Overlap
Oxidative Phosphorylation	9.49E-06	6/99 (6.1%)
Mitochondrial Dysfunction	1.36E-04	6/159 (3.8%)
Calcium Signaling	4.1E-04	6/195 (3.1%)
G Beta Gamma Signaling	2.3E-03	4/114 (3.62%)
Caveolar-mediated Endocytosis Signaling	4.82E-03	3/69 (4.3%)
Granulocyte Adhesion and Diapedesis	7.46E-03	4/156 (2.6%)

* P values were generated when the overlapped DEGs involving a specific were compared with the genes involving the same pathway in the IPA random dataset.

Supplementary Figure S1. Full-length western blot images for CDK1, cyclin B1, MCM5, NRF2, PLK3 and GAPDH with 2-week WT and *Lmna*$^{-/-}$ mouse heart tissue lysates, corresponding to the images in Figure 3c. Figures S1 a-f are listed in the next three pages.

a. CDK1 blot showed down-regulated CDK1 in *Lmna*$^{-/-}$ mice compared to that in WT mice;

b. Down-regulated cyclin B1 in *Lmna*$^{-/-}$ mice;

c. Down-regulated MCM5 in *Lmna*$^{-/-}$ mice;

d. Up-regulated NRF2 in *Lmna*$^{-/-}$ mice;

e. Up-regulated PLK3 in *Lmna*$^{-/-}$ mice;

f. GAPDH blot (as loading control).

a

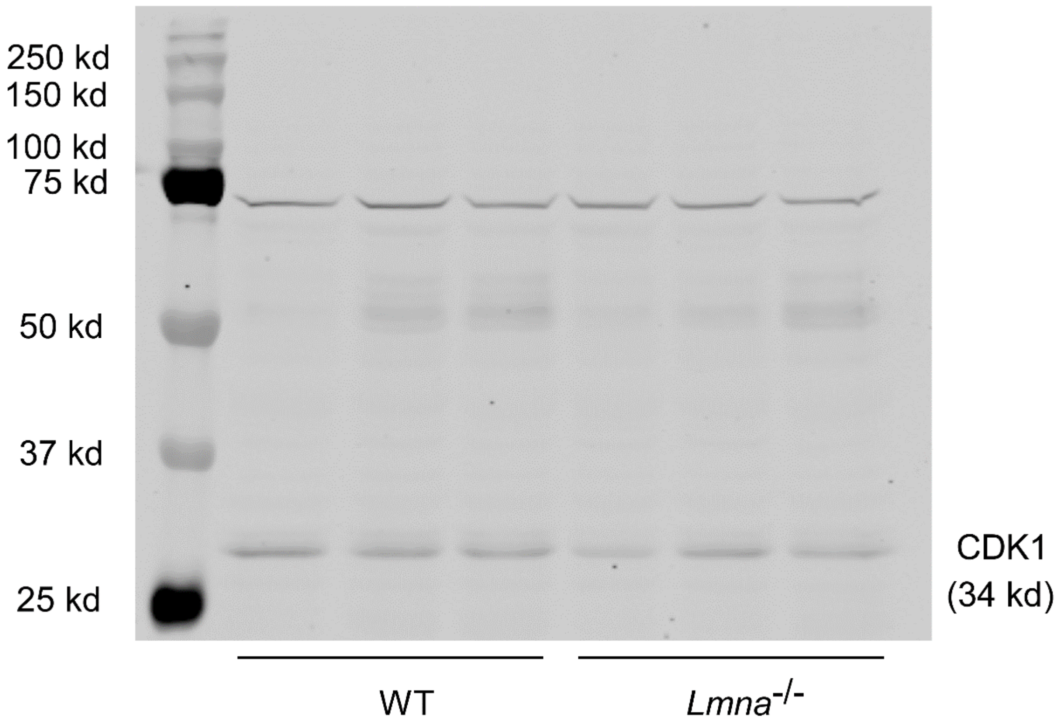
250 kd
150 kd
100 kd
75 kd
50 kd
37 kd
25 kd
CDK1
(34 kd)
WT
Lmna-/-

b

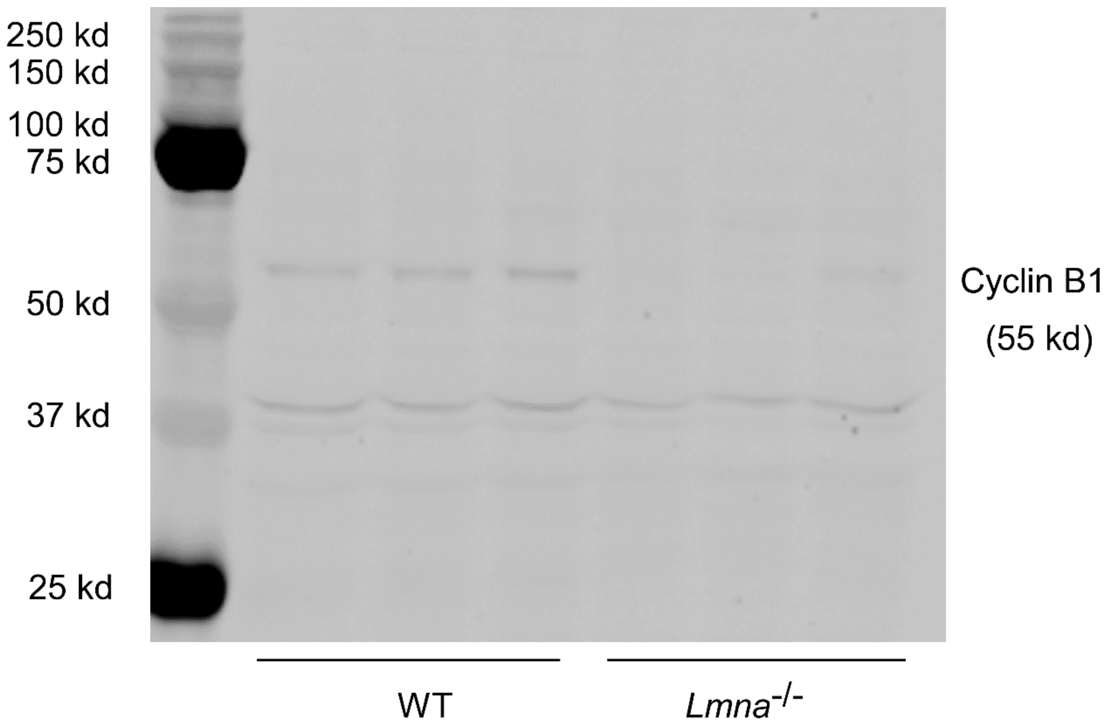
250 kd
150 kd
100 kd
75 kd
50 kd
37 kd
25 kd
Cyclin B1
(55 kd)
WT
Lmna-/-

c

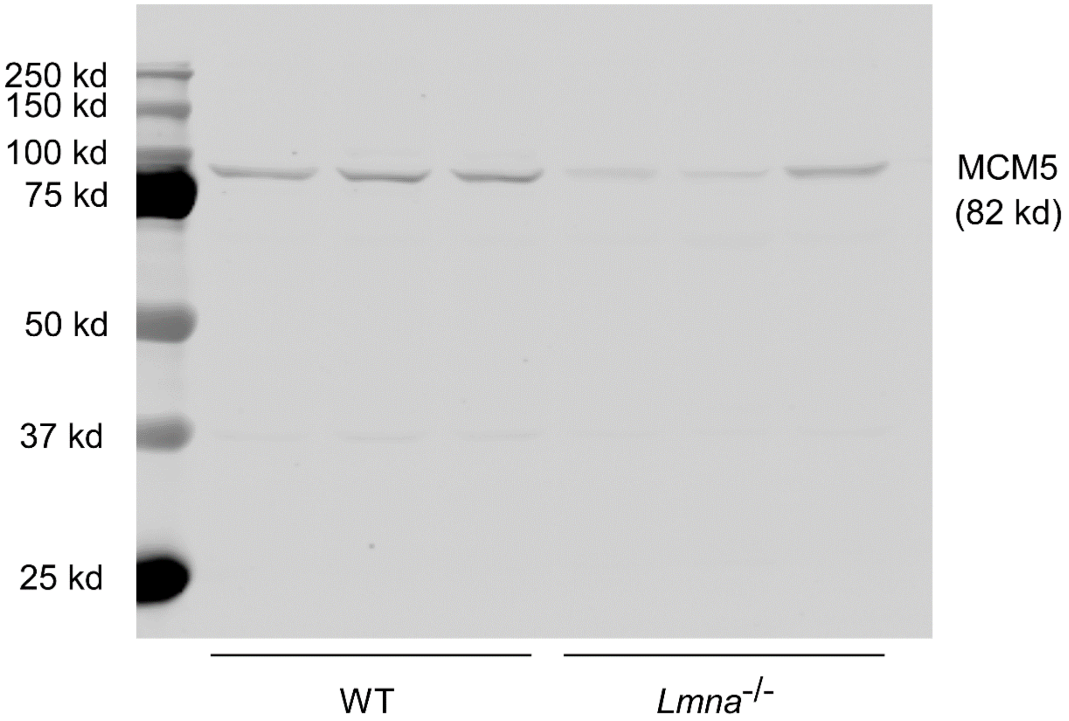
250 kd
150 kd
100 kd
75 kd
50 kd
37 kd
25 kd
MCM5
(82 kd)
WT
Lmna-/-

d

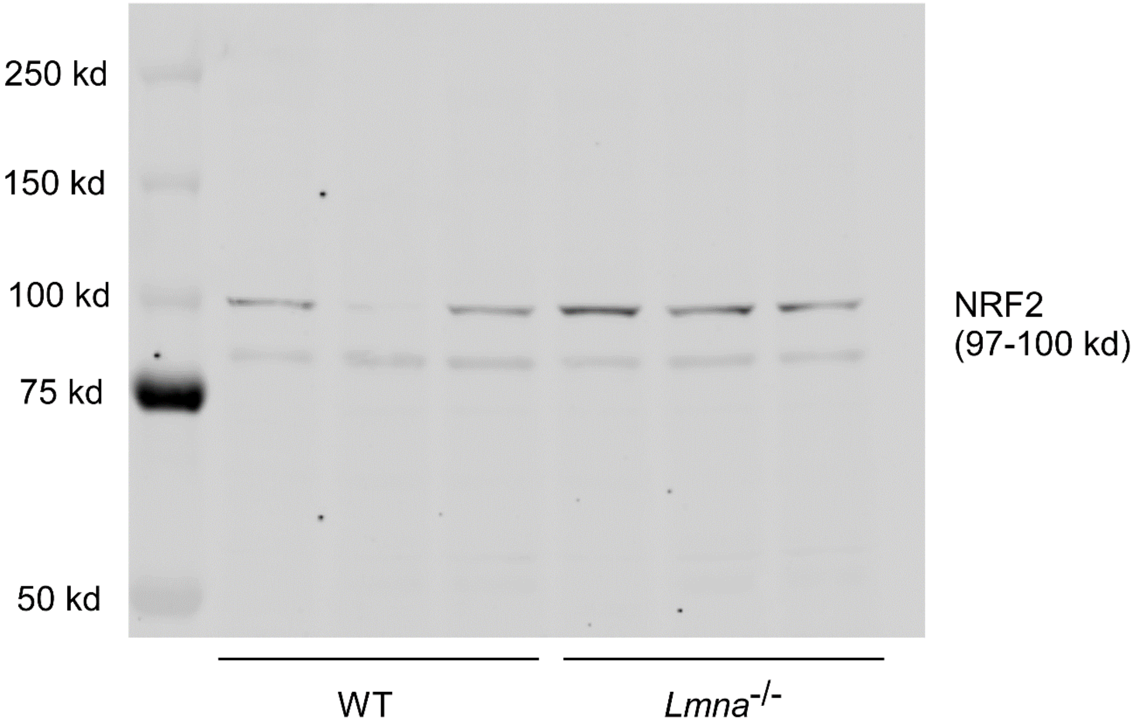
250 kd
150 kd
100 kd
75 kd
50 kd
NRF2
(97-100 kd)
WT
Lmna-/-

e

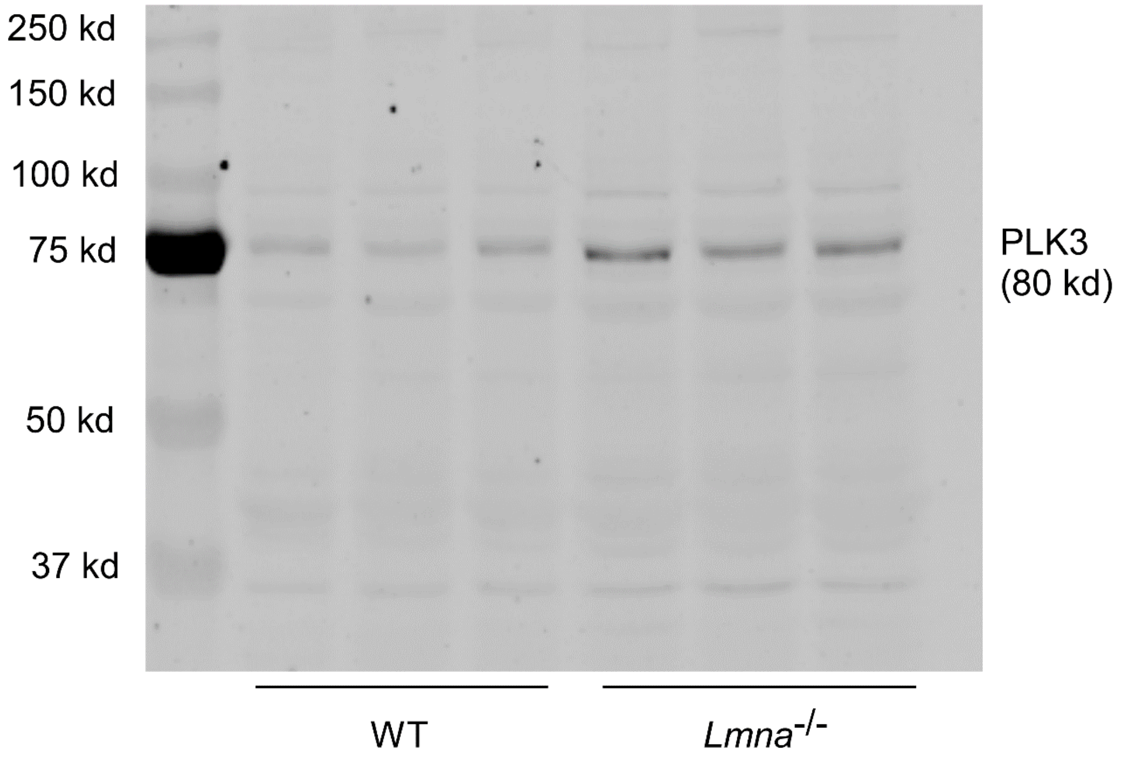
250 kd
150 kd
100 kd
75 kd
50 kd
37 kd
PLK3
(80 kd)
WT
Lmna-/-

f

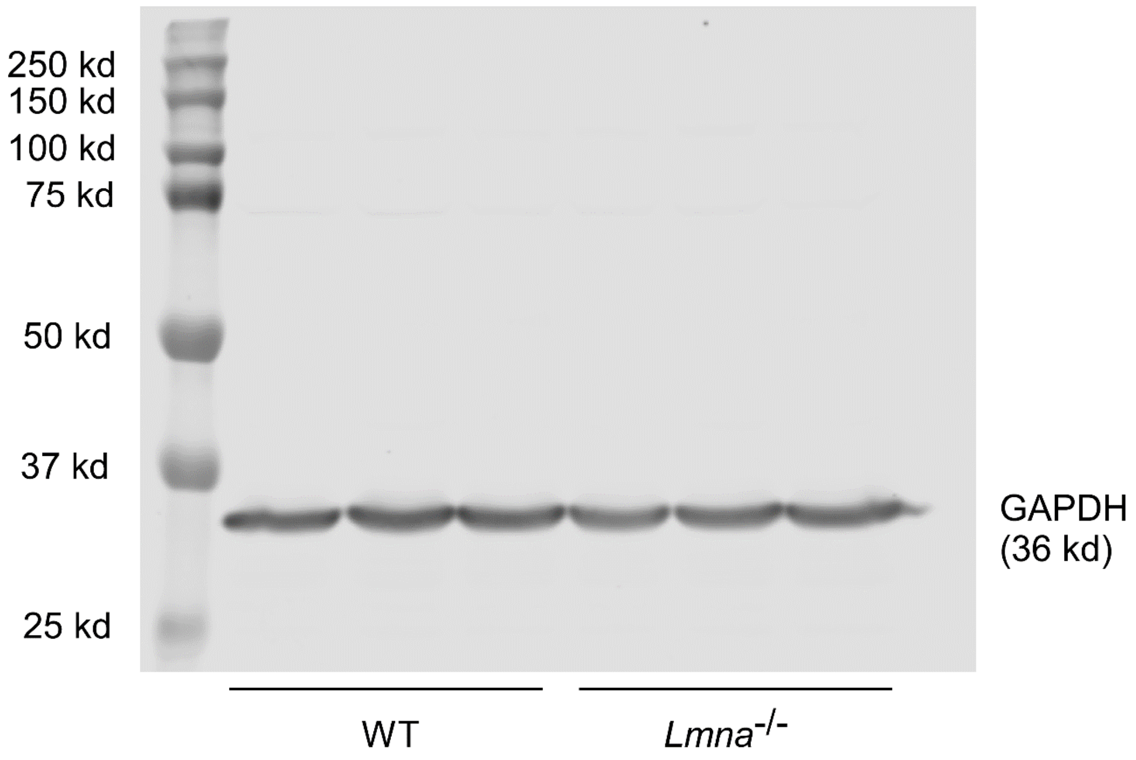
250 kd
150 kd
100 kd
75 kd
50 kd
37 kd
25 kd
GAPDH
(36 kd)
WT
Lmna-/-

Supplementary Figure S2. Full-length western blot images for ECH1,PPARA, β-actin, DUSP4, FHL1 and GAPDH with 1-month WT and *Lmna*$^{-/-}$ mouse heart tissue lysates, corresponding to the images in Figure 4c. Figures S2 a-f are listed in the next three pages.

a. ECH1 blot showed down-regulated ECH1 in *Lmna*$^{-/-}$ mice compared to that in WT mice;

b. Down-regulated PPARA in *Lmna*$^{-/-}$ mice;

c. β-actin blot (as loading control for ECH1 and PPARA);

d. Up-regulated DUSP4 in *Lmna*$^{-/-}$ mice;

e. Up-regulated FHL1 in *Lmna*$^{-/-}$ mice;

f. GAPDH blot (as loading control for DUSP4 and FHL1).

a

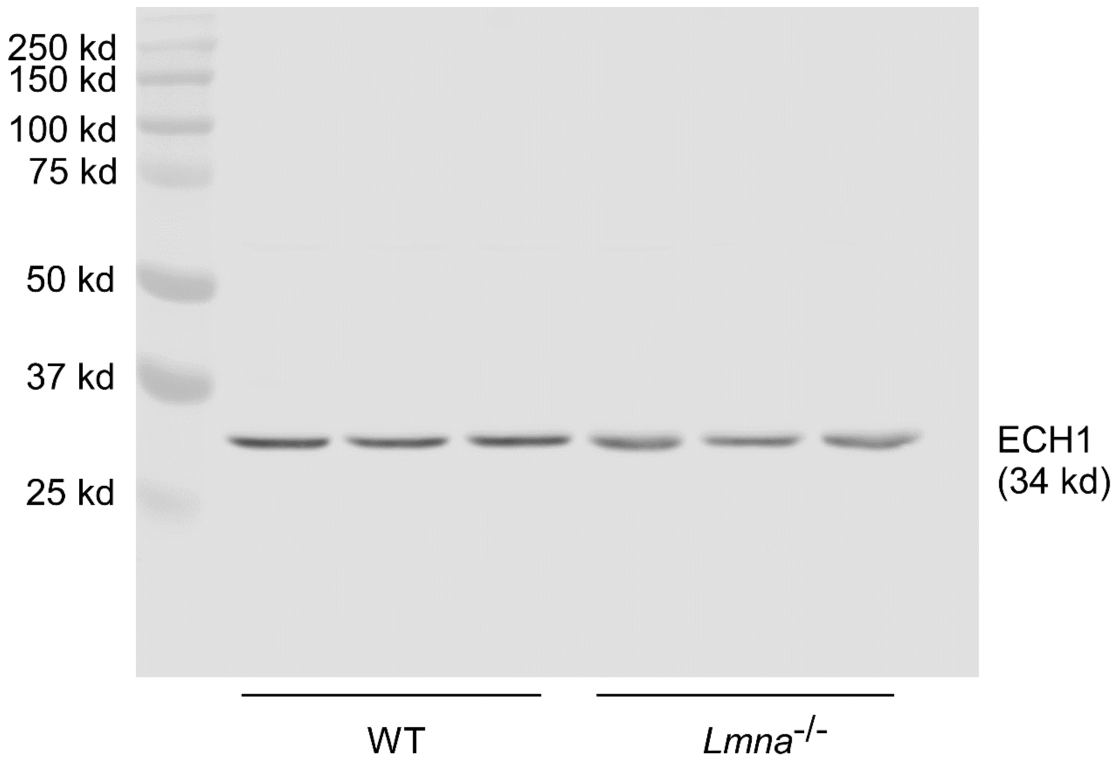
250 kd
150 kd
100 kd
75 kd
50 kd
37 kd
25 kd
ECH1
(34 kd)
WT
Lmna-/-

b

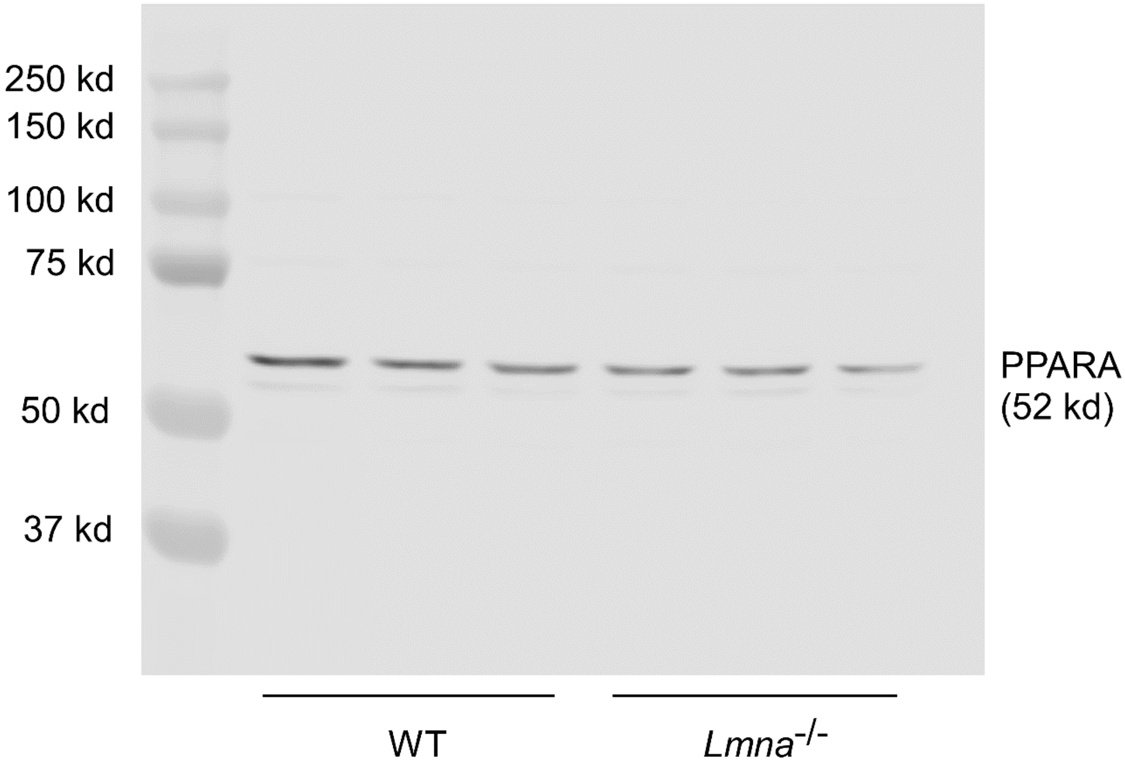
250 kd
150 kd
100 kd
75 kd
50 kd
37 kd
PPARA
(52 kd)
WT
Lmna-/-

c

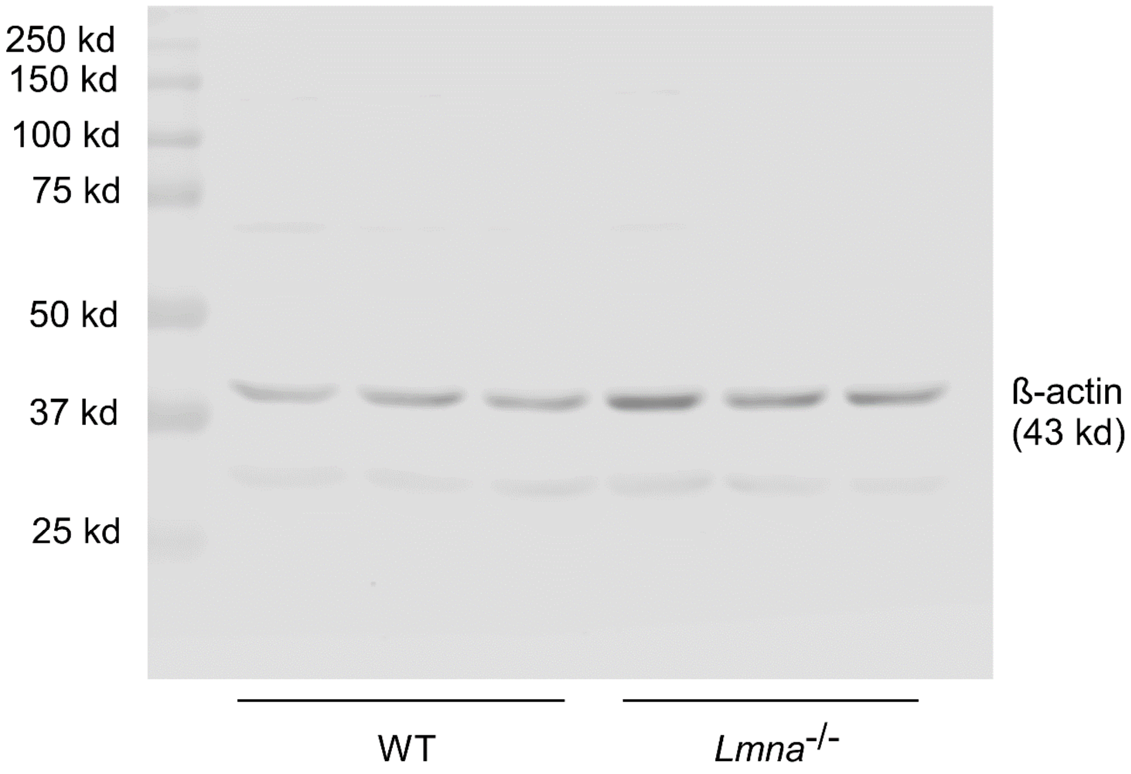
250 kd
150 kd
100 kd
75 kd
50 kd
37 kd
25 kd
ß-actin
(43 kd)
WT
Lmna-/-

d

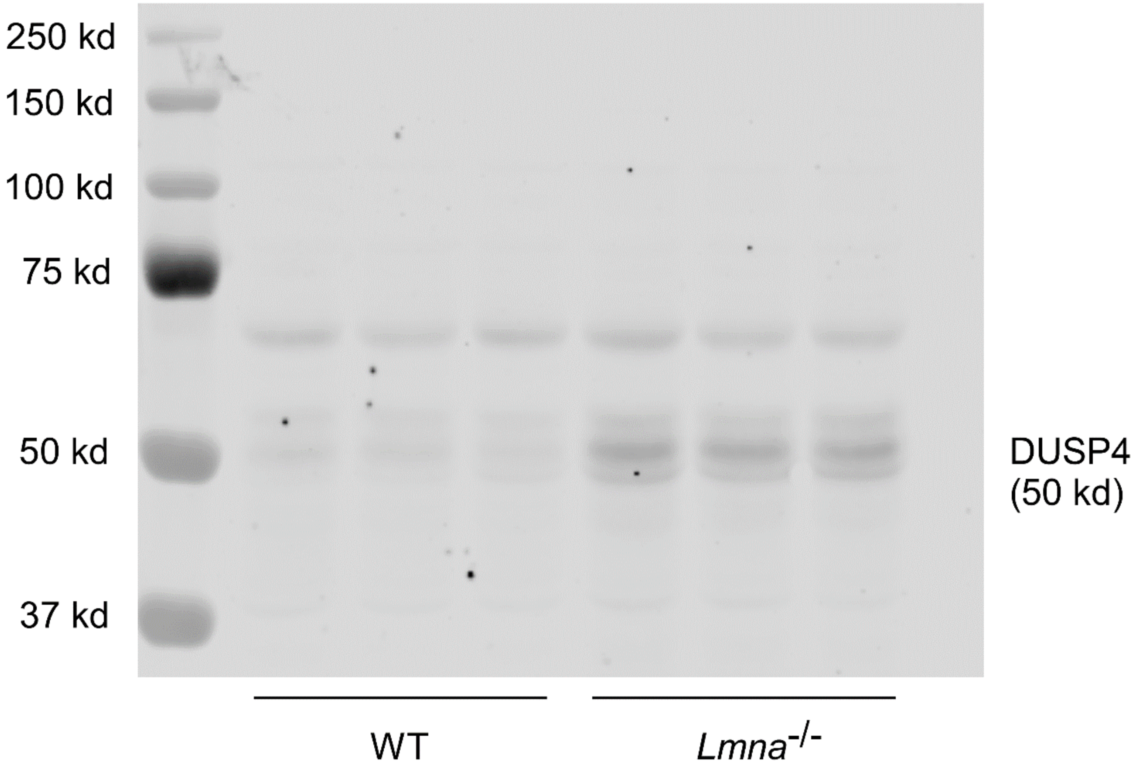
250 kd
150 kd
100 kd
75 kd
50 kd
37 kd
DUSP4
(50 kd)
WT
Lmna-/-

e

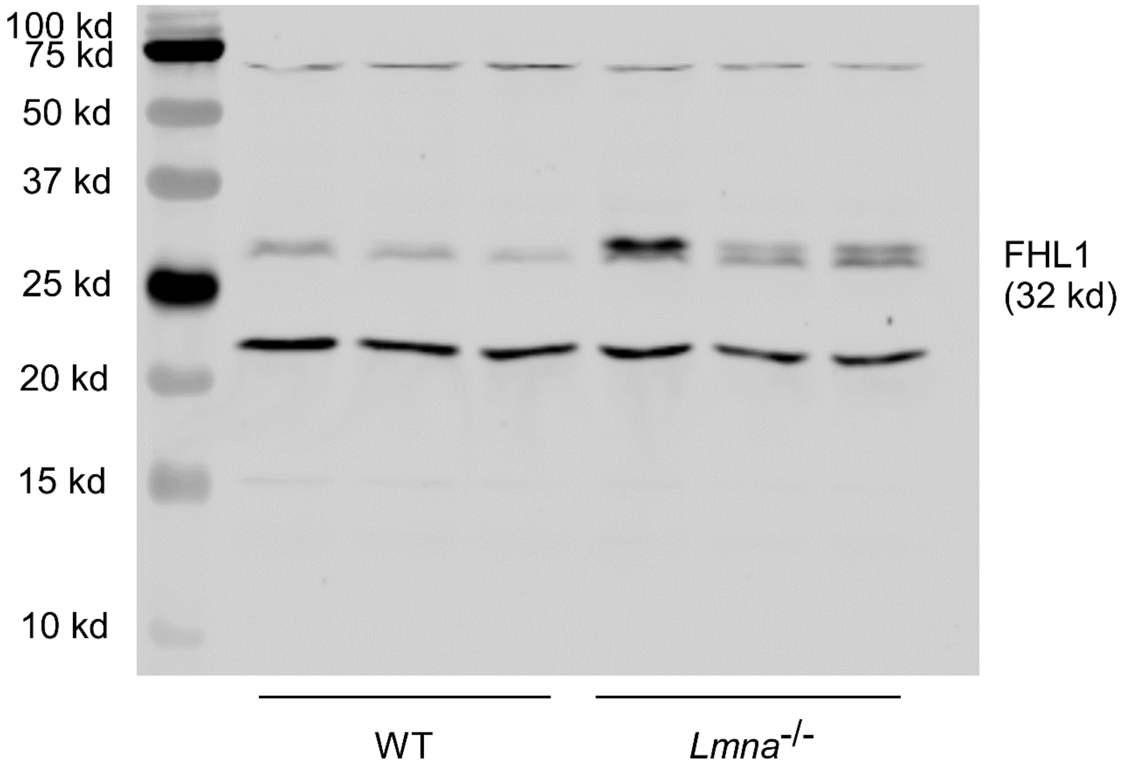
100 kd
75 kd
50 kd
37 kd
25 kd
20 kd
15 kd
10 kd
FHL1
(32 kd)
WT
Lmna-/-

f

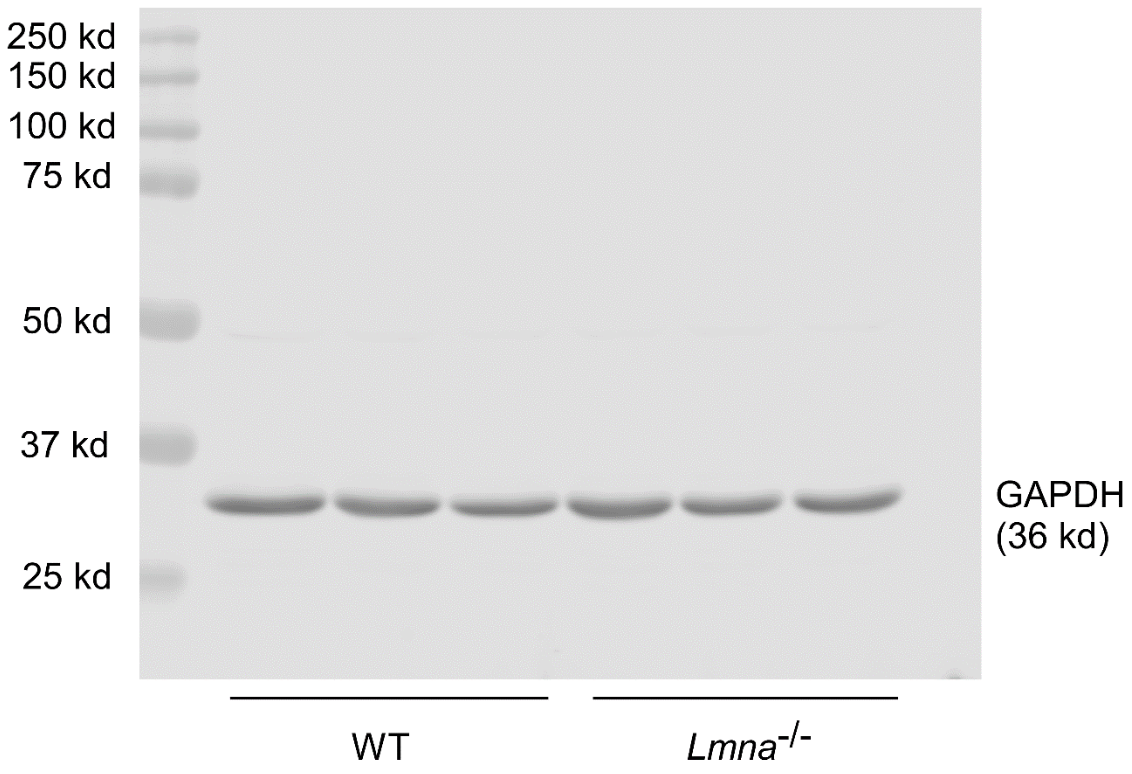
250 kd
150 kd
100 kd
75 kd
50 kd
37 kd
25 kd
GAPDH
(36 kd)
WT
Lmna-/-
